# Asia‐Pacific Consensus Recommendations on X‐Linked Hypophosphatemia: Diagnosis, Multidisciplinary Management, and Transition From Pediatric to Adult Care

**DOI:** 10.1002/jbm4.10744

**Published:** 2023-05-01

**Authors:** Craig F Munns, Han‐Wook Yoo, Muhammad Yazid Jalaludin, Rashida Vasanwala, Manju Chandran, Yumie Rhee, Wai Man BUT, Alice Pik‐Shan Kong, Pen‐Hua Su, Nawaporn Numbenjapon, Noriyuki Namba, Yasuo Imanishi, Roderick J Clifton‐Bligh, Xiaoping Luo, Weibo Xia

**Affiliations:** ^1^ Child Health Research Centre The University of Queensland Brisbane Australia; ^2^ Department of Endocrinology and Diabetes Queensland Children's Hospital Brisbane Australia; ^3^ Department of Pediatrics Bundang CHA Medical Center, CHA University School of Medicine Seongnam‐si Korea; ^4^ Department of Pediatrics Faculty of Medicine, Universiti Malaya Kuala Lumpur Malaysia; ^5^ Department of Paediatric Medicine Endocrine & Diabetes Service, KK Women's & Children's Hospital Singapore Singapore; ^6^ Osteoporosis and Bone Metabolism Unit, Department of Endocrinology, Singapore General Hospital Singapore Singapore; ^7^ Department of Internal Medicine Yonsei University College of Medicine Seoul Korea; ^8^ Department of Pediatrics Queen Elizabeth Hospital Hong Kong; ^9^ Department of Medicine & Therapeutics The Chinese University of Hong Kong Hong Kong; ^10^ Department of Pediatrics Chung Shan Medical University Hospital Taichung Taiwan; ^11^ School of Medicine Chung Shan Medical University Taichung Taiwan; ^12^ Division of Endocrinology, Department of Pediatrics Phramongkutklao Hospital Bangkok Thailand; ^13^ Department of Pediatrics and Perinatology Faculty of Medicine, Tottori University Tottori Japan; ^14^ Department of Metabolism Endocrinology and Molecular Medicine, Osaka Metropolitan University Graduate School of Medicine Osaka Japan; ^15^ Department of Endocrinology Royal North Shore Hospital, University of Sydney Sydney Australia; ^16^ Department of Pediatrics Tongji Hospital, Tongji Medical College, Huazhong University of Science and Technology Wuhan China; ^17^ Department of Endocrinology, Key Laboratory of Endocrinology, NHC, State Key Laboratory of Complex Severe and Rare Diseases Peking Union Medical College Hospital, Chinese Academy of Medical Sciences & Peking Union Medical College Beijing China

**Keywords:** X‐LINKED HYPOPHOSPHATEMIC RICKETS, CARE TRANSITION, PRACTICE GUIDELINES, ASIA, ASIA‐PACIFIC

## Abstract

X‐linked hypophosphatemia (XLH) is a rare, inherited, multisystem disorder characterized by hypophosphatemia that occurs secondary to renal phosphate wasting. Mutations in PHEX gene (located at Xp22.1) in XLH alter bone mineral metabolism, resulting in diverse skeletal, dental, and other extraskeletal abnormalities that become evident in early childhood and persist into adolescence and adult life. XLH impacts physical function, mobility, and quality of life, and is associated with substantial socioeconomic burden and health care resource utilization. As the burden of illness varies with age, an appropriate transition of care from childhood and adolescence to adulthood is necessary to meet growth‐related changes and minimize long‐term sequelae of the condition. Previous XLH guidelines that encompassed transition of care have focused on Western experience. Regional differences in resource availability warrant tailoring of recommendations to the Asia‐Pacific (APAC) context. Hence, a core expert panel of 15 pediatric and adult endocrinologists from nine countries/regions across APAC convened to formulate evidence‐based recommendations for optimizing XLH care. A comprehensive literature search on PubMed using MeSH and free‐text terms relevant to predetermined clinical questions on diagnosis, multidisciplinary management, and transition of care of XLH revealed 2171 abstracts. The abstracts were reviewed independently by two authors to shortlist a final of 164 articles. A total of 92 full‐text articles were finally selected for data extraction and drafting the consensus statements. Sixteen guiding statements were developed based on review of evidence and real‐world clinical experience. The GRADE criteria were used to appraise the quality of evidence supporting the statements. Subsequently, a Delphi technique was utilized to rate the agreement on statements; 38 XLH experts (15 core, 20 additional, 3 international) from 15 countries/regions (12 APAC, 3 EU) participated in the Delphi voting to further refine the statements. Statements 1–3 cover the screening and diagnosis of pediatric and adult XLH; we have defined the clinical, imaging, biochemical, and genetic criteria and raised red flags for the presumptive and confirmatory diagnosis of XLH. Statements 4–12 tackle elements of multidisciplinary management in XLH such as therapeutic goals and options, composition of the multidisciplinary team, follow‐up assessments, required monitoring schedules, and the role of telemedicine. Treatment with active vitamin D, oral phosphate, and burosumab is discussed in terms of applicability to APAC settings. We also expound on multidisciplinary care for different age groups (children, adolescents, adults) and pregnant or lactating women. Statements 13–15 address facets of the transition from pediatric to adult care: targets and timelines, roles and responsibilities of stakeholders, and process flow. We explain the use of validated questionnaires, desirable characteristics of a transition care clinic, and important components of a transfer letter. Lastly, strategies to improve XLH education to the medical community are also elaborated in statement 16. Overall, optimized care for XLH patients requires prompt diagnosis, timely multidisciplinary care, and a seamless transfer of care through the coordinated effort of pediatric and adult health care providers, nurse practitioners, parents or caregivers, and patients. To achieve this end, we provide specific guidance for clinical practice in APAC settings. © 2023 The Authors. *JBMR Plus* published by Wiley Periodicals LLC on behalf of American Society for Bone and Mineral Research.

## Graphical abstract

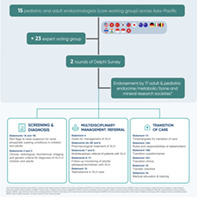

## Introduction

X‐linked hypophosphatemia (XLH) is a rare, genetic, multisystem disorder characterized by renal phosphate wasting. Mutations in the Phosphate Endopeptidase Homolog, X‐linked (PHEX) gene (located at Xp22.1) that encodes a transmembrane endopeptidase, primarily expressed in osteoblasts, osteocytes, and teeth, cause elevated fibroblast growth factor 23 (FGF23) and consequent renal phosphate wasting and defects in the hydroxylation of vitamin D. The altered mineral metabolism then leads to diverse skeletal abnormalities, including rickets, osteomalacia, and dental health issues.^(^ ^1^, ^2^, ^3^, ^4^, ^5^, ^6^, ^7^ ^)^

XLH is the most frequent cause of inherited hypophosphatemia and the most common genetic etiology of rickets with a prevalence of ~15–48 per 1 million individuals.^(^ ^8^, ^9^, ^10^ ^)^ In terms of the Asia‐Pacific (APAC) region, an incidence of 1 in 20,000 was reported in Japan.^(^ ^11^ ^)^ The symptoms and complications of XLH begin in early childhood, potentially leading to short stature and limb deformities. They may also extend into or develop further during adulthood, causing continued impairment of function, mobility, and quality of life (QoL), as well as increased health care resource utilization.^(^ ^12^, ^13^, ^14^ ^)^ In fact, adults with XLH may even have reduced survival compared with healthy controls.^(^ ^8^ ^)^

Burden‐of‐disease surveys in various parts of the world, including East Asia, reveal that despite conventional pharmacologic treatment with oral phosphate and active vitamin D, XLH imposes a lifelong disability from childhood to adulthood.^(^ ^15^, ^16^ ^)^ The higher prevalence of comorbidities in XLH patients, such as depression, other mental health problems, and neurologic conditions, inflicts additional debilitation throughout their life course.^(^ ^17^ ^)^ Furthermore, the burden of XLH and associated health care needs vary with age. In childhood, long‐term pharmacologic treatment may particularly be a burdensome issue to caregivers. Among adolescents, the psychological impact of XLH may become more apparent, while among adults, the need for surgery may emerge as a concern.^(^ ^18^ ^)^

Although there are international guidelines on XLH, not all of them provide clear guidance on transition of care. Moreover, the existing guidelines have so far focused only on the Western experience, which may not be applicable to the APAC region, where resource availability (ie, medications, diagnostic testing, and multidisciplinary expertise) may be different. Furthermore, in the majority of the current XLH guidelines, the lack of a validated methodology to obtain consensus recommendations constitutes another key unmet need.^(^ ^12^, ^13^, ^19^, ^20^, ^21^, ^22^, ^23^ ^)^

Hence, we aimed to use the Delphi methodology to develop the most up‐to‐date XLH consensus recommendations on the diagnosis, multidisciplinary management, and transition from pediatric to adult care, specifically tailored to the APAC setting.

## Methods

### APAC XLH Working Group

An APAC XLH Working Group was formed, composed of a core expert panel of 15 pediatric and adult endocrinologists from nine APAC countries/regions (Australia, China, Hong Kong, Japan, Malaysia, Singapore, South Korea, Taiwan, and Thailand). Meanwhile, a voting group composed of 38 adult and pediatric XLH experts (including 15 core group and 20 additional XLH experts from 12 APAC regions and 3 international XLH experts from the European Union) was also convened. In addition, the consensus recommendations were reviewed and endorsed by 17 local and regional endocrine, metabolic, and bone and mineral research societies, including the Asia Pacific Paediatric Endocrine Society (APPES), Australia and New Zealand Society for Paediatric Endocrinology and Diabetes (ANZSPED), the Australia and New Zealand Bone and Mineral Society (ANZBMS), the Chapter of Endocrinologists, College of Physicians (Singapore), the Chinese Society of Osteoporosis and Bone Mineral Research (CSOBMR), the Chinese Society of Paediatric Endocrinology and Metabolism (CSPEM), the Endocrine and Metabolic Society of Singapore (EMSS), the Hong Kong Society of Paediatric Endocrinology and Metabolism (HKSPEM), the Japanese Society for Bone and Mineral Research (JSBMR), the Japan Endocrine Society (JES), the Japanese Society for Pediatric Endocrinology (JSPE), the Korean Society for Bone and Mineral Research (KSBMR), the Korean Endocrine Society (KES), the Korean Society of Pediatric Endocrinology (KSPE), the Malaysian Endocrine and Metabolic Society (MEMS), the Taiwan Paediatric Association, and Taiwan Precision Children's Health Association.

### Literature search, review, and evidence rating

Initially, 16 research questions focusing on key aspects of XLH diagnosis, multidisciplinary management, and transition of care were drafted (Supplemental Table S1). To answer these questions, a comprehensive search was conducted on PubMed (MEDLINE) using a combination of relevant Medical Subject Headings (MeSH) and free‐text terms (Supplemental Table S2). Studies (randomized controlled trials [RCTs], systematic reviews, observational studies, surveys, case series, and case reports) published from January 2012 to September 2021, with abstracts in English and conducted in children and adults with XLH, were considered for inclusion. Two authors (CM and WX) independently reviewed the search screening results and the data extracted from the shortlisted studies. The quality of evidence was assessed using the GRADE criteria and GRADEpro guideline development tool and rated as very low, low, moderate, and high.^(^ ^24^, ^25^, ^26^, ^27^ ^)^

### Consensus building

Through an iterative editing process, preliminary draft statements were developed using the available evidence and subsequently presented and discussed by the APAC XLH Working Group members at an advisory meeting on November 20, 2021. During the meeting, the expert panel reviewed the literature and provided feedback to enable statement revision.

A Delphi technique was then applied to build consensus for each draft statement. A total of 38 XLH experts participated in two rounds of online voting, where the experts were asked to provide their agreement on the draft statement (on a scale of agree/disagree), with a provision to submit their comments for further refinement of the statements. Consensus was signified by an a priori agreement level of at least 70%.^(^ ^12^, ^28^ ^)^ The statements that received comments during the first online voting were modified and rated again in the second round of online voting. The strength of the consensus was defined as “strong” (>90% agreement), “moderate” (70%–90%), or “weak or no consensus” (<70%).

## Results

**Fig. 1:**
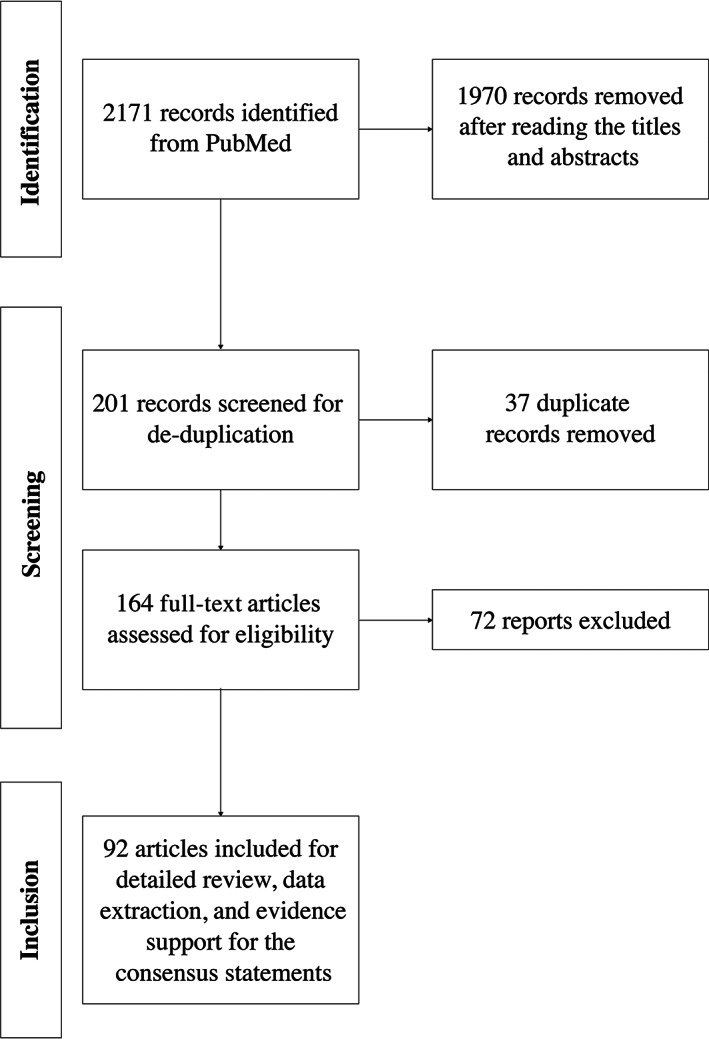
Flow diagram for article selection.

The literature search yielded a total of 2171 records (Fig. 1). After title and abstract screening and de‐duplication, 164 reports underwent full‐text review for eligibility. Finally, 92 reports were included and used to draft 16 consensus statements. The statements were centered around three themes: screening and diagnosis, multidisciplinary management, and transition from pediatric to adult care. A final statement on medical education and training was also provided.

The majority of the included studies had an observational design (mostly cross‐sectional). The rest were case reports or case series. The identified studies were similarly distributed among children and adults. In terms of outcomes, a high number of studies reported the prevalence of clinical, radiographic, and biochemical features of XLH, whereas outcomes of the management of XLH were less often reported. Studies from the APAC region constituted <15% of the identified reports, most of which came from China. As with the general set, the APAC studies were retrospective cohort or cross‐sectional studies, case reports, or case series.

Among the consensus statements, those on screening, diagnosis, and treatment of XLH had the largest body of high‐quality supporting evidence. Meanwhile, statements on multidisciplinary referrals, monitoring, and follow‐up were based on evidence of varying quality. The evidence supporting statements on the transition of XLH from childhood to adult care was limited. Nevertheless, the final statements reached a high percentage of consensus through the Delphi voting and were rated as strong recommendations (Table 1).

**Table 1:** Central Themes of the 16 Consensus Statements and Summary of Agreement Ratings From the Two Rounds of Delphi Voting

Consensus statement	Agreement rating on Delphi 1 (%)	Agreement rating on Delphi 2 (%)
Theme: Screening and diagnosis		
Statement 1A	84.21	97.37
Statement 1B	81.58	100
Statement 2A	76.32	94.74
Statement 2B	68.42	94.74
Statement 3A	76.32	92.11
Statement 3B	68.42	97.37
Theme: Multidisciplinary management		
Statement 4	92.11	100
Statement 5	81.58	97.37
Statement 6	94.74	100
Statement 7	89.47	100
Statement 8	71.05	100
Statement 9	68.42	100
Statement 10	78.95	97.37
Statement 11	92.11	100
Statement 12	100	–
Theme: Transition of care from childhood to adulthood		
Statement 13	89.47	100
Statement 14A	97.37	97.37
Statement 14B	94.74	100
Statement 14C	89.47	100
Statement 15	89.47	97.37
Theme: Education and training		
Statement 16	97.37	100

## Discussion

### Screening and diagnosis

Our recommendations for optimizing the screening and diagnosis of XLH in the APAC region are presented in Table 2 (Statements 1–3). A diagnosis of XLH is established via a combination of clinical, radiographic, biochemical, and genetic characteristics. The age of onset of clinical features of XLH in the majority of cases is between 1 and 2 years,^(^ ^29^, ^30^, ^31^, ^32^, ^33^, ^34^ ^)^ but diagnosis is established later between 2 and 3 years of age.^(^ ^35^, ^36^, ^37^, ^38^, ^39^, ^40^, ^41^, ^42^, ^43^, ^44^, ^45^ ^)^ This is because of the variable clinical phenotype of XLH, which may often lead to misdiagnosis or delay in diagnosis.^(^ ^23^, ^46^ ^)^ Early diagnosis of XLH leads to prompt treatment and better outcomes.^(^ ^36^, ^37^, ^40^, ^47^ ^)^ Hence, early recognition of renal phosphate wasting is essential (Table 2, Statements 1A and 1B).

**Table 2:** Asia‐Pacific Consensus Recommendations for Optimizing the Screening and Diagnosis of XLH

Statement 1A	GRADE evidence quality: ⨁⨁⨁◯ Moderate
Renal phosphate wasting conditions should be suspected and evaluated further in children with decreased growth velocity, short stature,^a^ and/or lower limb deformities with radiographic and/or clinical evidence of rickets.^b^ Additional red flags may include a history of recurrent clinically significant fragility fractures, abnormal (waddling) gait, impaired gross motor function, bone pain, abnormal head shape with frontal bossing or craniosynostosis, recurrent dental abscess, or a family history of rickets or phosphate wasting disorder.	

Statement 1B	GRADE evidence quality: ⨁⨁⨁◯ Moderate
Hereditary (or congenital) renal phosphate wasting conditions should be suspected and evaluated further in adults with low serum phosphate levels and short stature,^a^ radiographic evidence of early degenerative arthritis, osteoporosis, pseudofractures or enthesopathies, bone pain with a history of lower limb deformities, or history of vitamin D‐resistant rickets or corrective osteotomy. Additional red flags may include endodontic abnormalities such as periodontitis, dental abscess, or premature loss of permanent teeth, or a family history of rickets or phosphate wasting disorders.	

Statement 2A	GRADE evidence quality: ⨁⨁⨁⨁ High
The “essential” clinical and/or radiological features for a presumptive diagnosis of XLH in children should include the following: Decreased growth velocity^c^ Short stature^a^ ^,^ ^c^ Progressive lower limb (varus or valgus) deformities often combined with intoeing/out‐toeing^c^ Clinical and/or radiographic signs of active rickets in the knees and/or wrists that do not heal with ≥3 months of calcium and vitamin D treatment The “essential” biochemical criteria to confirm the diagnosis of XLH in children should include all of the following: Serum phosphate level below the age‐related reference range^d^ Renal phosphate wasting (assessed by calculating renal tubular reabsorption of phosphate in the fasting state [TmP/GFR and %TRP] based on urinary and serum phosphate and creatinine levels)—other renal reasons for phosphaturia should be excluded by evaluating for glucosuria, proteinuria, aminoaciduria, and excessive urinary excretion of bicarbonate Persistently elevated ALP levels above the age‐related reference range	

Statement 2B	GRADE evidence quality: ⨁⨁⨁⨁ High
The additional criteria to support or further confirm the diagnosis of XLH in children may include: Clinical: Abnormal (waddling) gait, abnormal head shape; frontal bossing; recurrent dental abscess; large head circumference; bone pain Imaging: Widened, cupped, flared, and frayed metaphyses or costochondral junctions (rachitic rosary); Harrison's groove; craniosynostosis (CT/MRI); and/or Chiari type 1 malformation (CT/MRI) Biochemical: Normal serum calcium levels, normal or mildly elevated PTH levels, normal 25‐hydroxy vitamin D, low or inappropriately normal 1,25‐dihydroxy vitamin D, and/or elevated or inappropriately normal FGF23 Genetic: A positive family history of XLH (X‐linked dominant inheritance) and/or detection of pathogenic *PHEX* gene mutations—genetic criteria will help further confirm the diagnosis of XLH.	

Statement 3A	GRADE evidence quality: ⨁⨁⨁⨁ High
The “essential” criteria to confirm the diagnosis of XLH in adults should include the following biochemical features: Serum phosphate level below the laboratory reference range^d^ Renal phosphate wasting (assessed by calculating renal tubular reabsorption of phosphate in the fasting state [TmP/GFR and %TRP] based on urinary and serum phosphate and creatinine levels)—other renal reasons for phosphaturia should be excluded by evaluating for glucosuria, proteinuria, aminoaciduria, and excessive urinary excretion of bicarbonate. Additional clinical and imaging features that may help further confirm the diagnosis include: Short stature^a^ Presence or history of pseudofractures and/or lower limb deformities	

Statement 3B	GRADE evidence quality: ⨁⨁⨁⨁ High
The “additional” criteria to support or further confirm the diagnosis of XLH in adults may include: Clinical: Bone pain, stiffness, reduced functional capacity (eg, as assessed by six‐minute walk test), and/or recurrent dental abnormalities such as periodontitis or dental abscess Imaging: Radiological signs of osteomalacia, early osteoarthritis of the spine, hip, or knees, and/or enthesopathies Biochemical: Normal serum calcium levels, normal 25‐hydroxy vitamin D and inappropriately normal or low 1,25‐dihydroxy vitamin D, normal or elevated ALP levels, normal or elevated PTH levels, and/or elevated or inappropriately normal FGF23 Genetic: A positive family history of XLH (X‐linked dominant inheritance) and/or detection of pathogenic *PHEX* gene mutations—genetic criteria will help further confirm the diagnosis of XLH.	

Abbreviation: ALP = alkaline phosphatase; CT = computed tomography; FGF23 = fibroblast growth factor 23; GRADE = Grading of Recommendations, Assessment, Development, and Evaluations; MRI = magnetic resonance imaging; PHEX = Phosphate Endopeptidase Homolog X‐linked; PTH = parathyroid hormone; TmP/GFR = tubular maximum reabsorption of phosphate per glomerular filtration rate; TRP = tubular reabsorption of phosphate; XLH = X‐linked hypophosphatemia.

^a^ Disproportionate short stature in severe cases (impaired limb growth with preserved trunk growth).

^b^ Radiographic and/or clinical rickets that does not heal even after 3 months of vitamin D and calcium supplementation—for APAC regions with moderate‐to‐high prevalence of nutritional rickets.

^c^ Primarily in children who have started to walk (>1–1.5 years of age).

^d^ If clinical suspicion exists and serum phosphate levels are normal, “fasting” serum phosphate test should be conducted.

#### Clinical and radiologic features

The most common initial manifestations of XLH include short stature, bone deformities, and radiologic signs of rickets^(^ ^29^, ^30^, ^31^, ^32^, ^34^, ^35^, ^39^ ^)^ (Table 2, Statement 2A). These clinical and radiologic features should prompt timely and appropriate referral for biochemical testing, early confirmation of the diagnosis, and initiation of treatment. Genetic testing is not mandatory to confirm the diagnosis, given the resource constraints in some APAC countries.

Reduced growth velocity and short stature (ie, height *Z*‐score below −2 standard deviations) are found in 33%–92% of children with XLH.^(^ ^35^, ^36^, ^38^, ^39^, ^40^, ^41^, ^42^, ^43^, ^48^ ^)^ Although children with XLH are born with average length, growth velocity begins to deviate from the norm at 6 months of age, and reduced growth is evident by 1 year and persists throughout childhood until final height.^(^ ^32^, ^49^ ^)^ Short stature is thus observed in more than 50% (56%–95%) of adults with XLH.^(^ ^36^, ^42^, ^50^, ^51^, ^52^ ^)^ The short stature found in XLH may be disproportionate; specifically, leg length is shorter than trunk, leading to a substantially increased sitting height index.^(^ ^45^, ^52^ ^)^ The short stature in XLH may be misdiagnosed in some cases as achondroplasia, the most common form of skeletal dysplasia.^(^ ^46^ ^)^

The majority of patients with XLH have lower limb deformities.^(^ ^32^, ^33^, ^34^, ^36^, ^39^, ^40^, ^41^, ^43^, ^53^ ^)^ Genu/coxa varum or valgum is observed in more than 90% of most cohorts.^(^ ^32^, ^36^, ^39^, ^41^, ^53^ ^)^ These deformities may be associated with intoeing or an unstable or abnormal gait and cause delayed onset, difficulty, or pain with walking and early fatigue.^(^ ^29^, ^30^, ^32^, ^33^, ^54^, ^55^, ^56^ ^)^ Radiographic evidence of rickets is observed in 100% of XLH cohorts before treatment and in more than 70%, despite conventional treatment.^(^ ^32^, ^37^, ^38^, ^39^, ^41^ ^)^ Typical findings on skeletal X‐ray include widened and cupped metaphyses found at the distal end of the radius, ulna, femur, and tibia.^(^ ^32^, ^41^, ^57^, ^58^ ^)^ The major differential diagnosis for XLH is nutritional rickets that occurs due to vitamin D and/or dietary calcium deficiency.^(^ ^12^, ^54^, ^55^ ^)^ The severity, chronicity, progression, or persistence of signs and symptoms despite vitamin D and calcium supplementation may help differentiate between the two conditions and suggest XLH as the more likely diagnosis.^(^ ^12^, ^54^, ^55^ ^)^

Dental pathology (eg, gingivitis, periodontitis, dental malposition, dental abscess) is common among children with XLH and has been observed in up to 100% of cases.^(^ ^33^, ^35^, ^38^, ^40^, ^43^, ^52^, ^53^, ^59^, ^60^ ^)^ In XLH, dental abscesses are often recurrent and may be associated with fistula formation and occur without dental decay or history of injury.^(^ ^38^, ^43^ ^)^ Patients with XLH may present with delayed eruption of dentition or premature loss of teeth.^(^ ^29^, ^30^, ^32^, ^56^, ^61^ ^)^ Early loss of dentition and severe dental caries are also noted in hypophosphatasia, a rare inherited metabolic disorder. Differential diagnosis should be considered based on biochemical findings; hypophosphatasia is associated with reduced serum and bone alkaline phosphatase (ALP) activity,^(^ ^62^ ^)^ whereas XLH in children is associated with persistently elevated ALP levels.^(^ ^12^ ^)^

Other skeletal signs in children with XLH include enlarged wrists (46%), rachitic rosary (9% in treated cases and up to 38% in untreated cases), rib eversion (60%), pectus carinatum (47.7%), bracelet signs (64.6%), and skull malformations (7.9%–54%).^(^ ^33^, ^39^, ^41^, ^43^, ^63^ ^)^ The latter include frontal bossing, square head, scaphocephaly, and craniosynostosis.^(^ ^29^, ^32^, ^43^, ^53^, ^63^, ^64^ ^)^ Specifically, craniosynostosis may be an early sign of XLH in infancy, presenting with an abnormal head shape.^(^ ^64^, ^65^ ^)^ About 10% of children with XLH also have a concomitant Chiari malformation,^(^ ^43^ ^)^ characterized by extension of the brain tissue through the foramen magnum. Although most cases are asymptomatic, compression of the lower brain stem and upper spinal cord may cause symptoms, including severe headache, neck pain, and myriad motor, sensory, respiratory, and neurological complications that may result in syringomyelia, necessitating surgery.^(^ ^12^, ^13^, ^23^ ^)^ Aside from the cranium, bony affectation can also lead to the formation of Harrison's groove/sulcus at the lower end of the rib cage, whereas rachitic rosary develops at the costochondral junctions.^(^ ^12^, ^66^ ^)^

In adults with XLH, pseudofractures are noted in 29%–52% of cases.^(^ ^14^, ^51^, ^59^ ^)^ Other musculoskeletal manifestations frequently found in adults are enthesopathies (33%–100%) and osteoarthritis (55%–80%), which become more prevalent beyond 30–40 years of age.^(^ ^14^, ^37^, ^51^, ^52^, ^59^, ^67^ ^)^ Accordingly, pain is reported in almost 100% of adults with XLH and muscle weakness in 60% of cases, along with joint stiffness and impaired mobility or physical function (eg, using the 6‐minute walk test [6MWT]).^(^ ^14^, ^16^, ^23^, ^67^ ^)^ Dental abscesses are reported in about 82% of adults with XLH.^(^ ^23^ ^)^ Moreover, most adults with XLH (~50%–79%) have a history of orthopedic surgery, most commonly long bone osteotomy.^(^ ^14^, ^37^, ^51^, ^52^, ^53^, ^59^ ^)^

#### Biochemical features

Hypophosphatemia with renal phosphate wasting is a biochemical hallmark of XLH. Regardless of age, almost all XLH patients (95%–100%) display low serum phosphate levels.^(^ ^32^, ^36^, ^37^, ^38^, ^41^, ^42^, ^44^, ^60^ ^)^ However, hypophosphatemia may not be apparent in the first 3–6 months of life and may be missed because of varied age‐specific reference values and normal phosphate levels in some infants.^(^ ^12^, ^19^, ^23^ ^)^ Renal phosphate wasting is ideally assessed by determining the tubular maximum reabsorption of phosphate per glomerular filtration rate (TmP/GFR). Age‐related reference ranges are available for this parameter, which is computed from fasted, second‐morning, paired plasma and urine phosphate and creatinine levels.^(^ ^68^, ^69^, ^70^, ^71^ ^)^ The TmP/GFR is reduced in almost 100% of XLH patients.^(^ ^38^, ^42^, ^44^ ^)^ Nevertheless, XLH should be differentiated from non‐selective causes of renal phosphate wasting (ie, Fanconi syndrome) that present with other distinctive features such as bicarbonate and uric acid wasting, glucosuria, aminoaciduria, and low‐molecular‐weight proteinuria.^(^ ^12^, ^13^, ^19^, ^22^, ^23^ ^)^

Alkaline phosphatase, a marker of bone turnover, is elevated in association with rickets and osteomalacia. Because bone‐specific ALP constitutes ~90% of total ALP in children and only ~50% in adults, total ALP may be used for children, whereas bone‐specific ALP is recommended for adults.^(^ ^12^, ^13^, ^19^, ^22^, ^23^ ^)^ In some settings, bone‐specific ALP may not be available or commonly used.^(^ ^13^ ^)^ If the assay for bone‐specific ALP is not available, the traditional heat fractionation of ALP may be used.^(^ ^72^, ^73^ ^)^ As with phosphate levels, ALP follows an age‐based reference range, and though it may be normal in the first few months of life in some patients with XLH,^(^ ^13^, ^74^ ^)^ elevated ALP is demonstrated by 83%–100% of children (over 1 year of age) with XLH.^(^ ^32^, ^37^, ^38^, ^41^, ^42^ ^)^

The other biochemical parameters observed in most patients with XLH and their reported prevalence rates are normal serum calcium levels (89.2%–100%),^(^ ^37^, ^41^, ^42^, ^60^ ^)^ normal serum vitamin D levels (81.3%–100%),^(^ ^38^, ^41^ ^)^ normal or mildly increased serum parathyroid hormone (PTH) levels (54.4%–100%),^(^ ^33^, ^41^, ^42^ ^)^ and inappropriately normal or elevated intact fibroblast growth factor‐23 (iFGF‐23) levels (74.2%–100%).^(^ ^32^, ^33^, ^36^, ^37^ ^)^ iFGF‐23 testing is not widely available, and results should be interpreted with caution because of lack of standardization and potential influence by treatment.^(^ ^4^, ^11^ ^)^

25‐Hydroxy vitamin D levels should be normal in XLH, unless affected by factors such as sun exposure, nutrition, maternal vitamin D status, and comorbid renal conditions, although the levels of the active form, 1,25‐dihydroxyvitamin D, may be inappropriately low in the setting of hypophosphatemia.^(^ ^38^, ^41^ ^)^ Notably, in Asian cohorts, because of the overall high prevalence of nutritional rickets due to vitamin D deficiency, XLH patients may also have concurrent vitamin D deficiency (43.6%–59.8%), which may in turn mask the diagnosis of XLH.^(^ ^33^, ^75^ ^)^ With regard to PTH levels, XLH patients have higher levels versus healthy controls, although when compared with calcium‐deficient rickets, these levels are still within the upper bounds of normal or only slightly elevated.^(^ ^12^, ^44^ ^)^

#### Genetic features

Genetic testing confirms the diagnosis of XLH and facilitates genetic counseling of the patient and family members.^(^ ^30^, ^31^ ^)^ Single‐gene *PHEX* sequencing suffices as the first step in genetic analysis because it detects the most common cause of hypophosphatemic rickets.^(^ ^76^ ^)^ A confirmed pathogenic *PHEX* mutation is found in ~90%–100% of clinically diagnosed patients.^(^ ^37^, ^53^ ^)^ In case genetic testing is not available, a positive family history of XLH is generally accepted as supportive of the diagnosis.^(^ ^53^ ^)^ About 21%–82% of XLH patients have a family history of hypophosphatemic rickets and may be identified via family screening.^(^ ^35^, ^37^, ^39^, ^40^, ^43^, ^44^, ^53^ ^)^ The transmission consistent with XLH is X‐linked dominant inheritance: from male parent to female offspring or from female parent to 50% of male/female offspring. No genotype–phenotype correlation has been established.^(^ ^32^, ^33^, ^40^, ^60^, ^75^ ^)^

### Multidisciplinary management

**Fig. 2:**
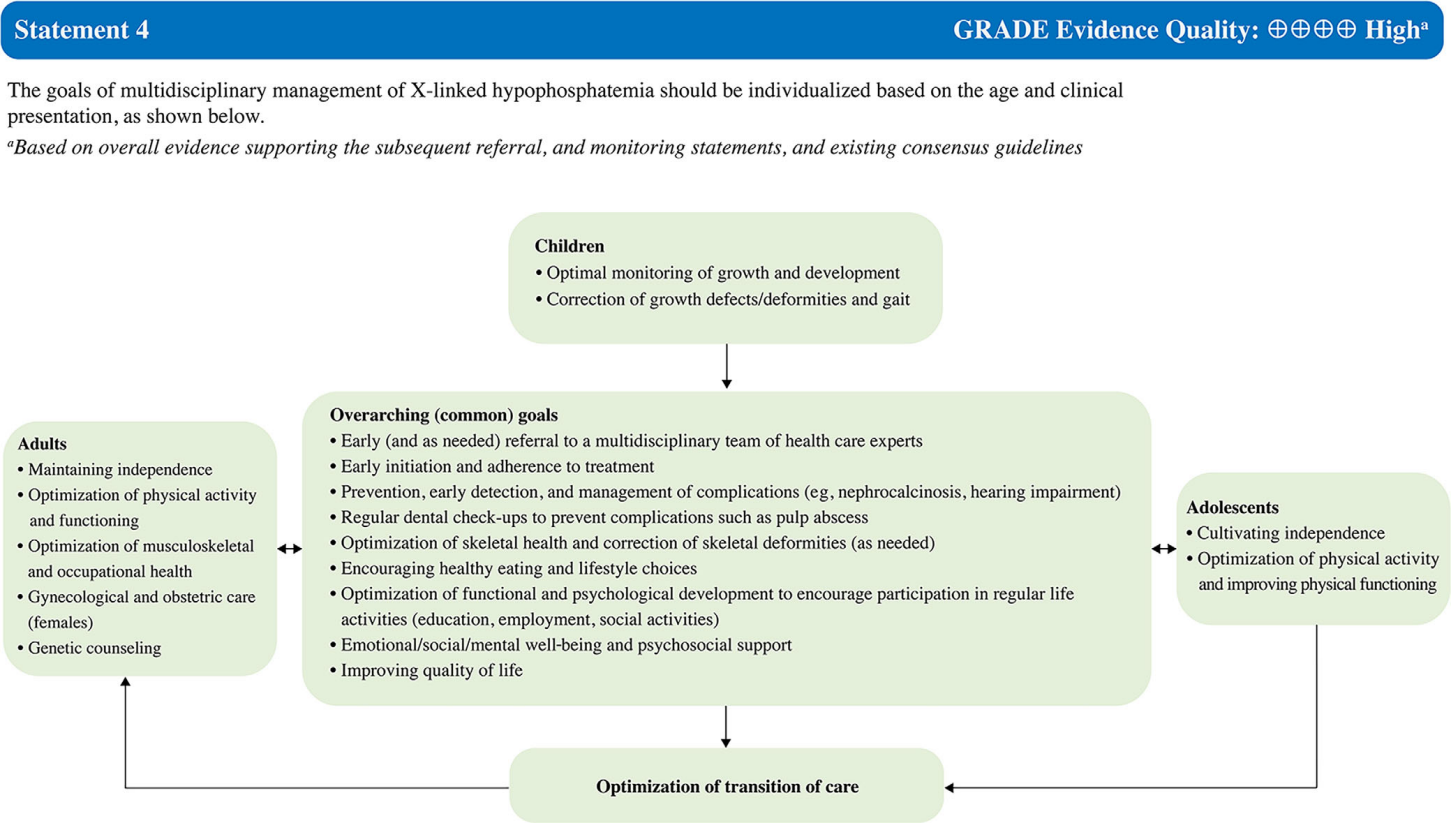
Asia‐Pacific consensus recommendations on goals of multidisciplinary management of X‐linked hypophosphatemia (XLH).

Our recommendations for optimizing the multidisciplinary management of XLH in the APAC region are shown in Fig. 2 (Statement 4) and Tables 3, 4, 5, 6, 7, 8 (Statements 5–12). The multitude of clinical, radiographic, and biochemical manifestations of XLH warrant multidisciplinary care. The goals of management should be tailored to the clinical presentation and the patient's age (Fig. 2, Statement 4).^(^ ^12^, ^17^, ^18^, ^77^ ^)^

**Table 3:** Asia‐Pacific Consensus Recommendations for the Pharmacologic Treatment of XLH

Statement 5A	GRADE evidence quality: ⨁⨁⨁⨁ High
Children and symptomatic adults with XLH should be treated with a combination of oral phosphate and active vitamin D (calcitriol or alfacalcidol) according to international or national guidelines. The benefits and side effects of conventional oral phosphate plus active vitamin D should be discussed with the patient before initiating treatment.	

Statement 5B	GRADE evidence quality: ⨁⨁⨁⨁ High
Children and adults with XLH on treatment with conventional active vitamin D and oral phosphate should be monitored closely for prevention and early management of associated complications such as gastrointestinal discomfort, hyperparathyroidism, hypercalciuria, hypercalcemia, renal insufficiency, or nephrocalcinosis/nephrolithiasis.	

Statement 5C	Evidence quality: Expert opinion
Accessibility to appropriate optimal forms of phosphate salts and/or active vitamin D is a major issue in several APAC regions. All APAC countries should have access to appropriate forms of oral phosphate (or pharmaceutical‐grade phosphate) and active vitamin D for early and optimized management of XLH and prevention of XLH‐associated complications.	

Statement 5D	GRADE evidence quality: ⨁⨁⨁⨁ High
Treatment with burosumab should be initiated, if available, in children aged ≥1 year, adolescents, and adults with XLH, and monitored according to indications and guidance provided in the product prescribing information.	

Statement 5E	Evidence quality: Expert opinion
Accessibility to burosumab should be improved in APAC settings to enable optimization of XLH management, improvement in functional and patient‐reported outcomes and quality of life, and prevention of XLH‐associated complications, including fractures.	

Statement 6	Evidence quality: Expert opinion
Pregnant and lactating women with XLH may be treated with active vitamin D in combination with oral phosphate supplements, if needed. However, they should be monitored more frequently (at least once every 3 months) for complications. Burosumab is currently not recommended during pregnancy. It is unknown whether burosumab or its metabolites are excreted in human milk.	

Abbreviation: APAC = Asia‐Pacific; GRADE = Grading of Recommendations, Assessment, Development, and Evaluations; XLH = X‐linked hypophosphatemia.

**Table 4:** Asia‐Pacific Consensus Recommendations for Multidisciplinary Referral of Patients With XLH

Statement 7	GRADE evidence quality
We recommend that, if resources are available, a child with suspected or confirmed XLH should be referred to and followed up by a multidisciplinary team composed of:	
A physician with special interest in XLH—often a pediatric endocrinologist but in some settings a pediatric nephrologist or a general pediatrician—for the medical treatment of metabolic bone disease and other complications (eg, pain) associated with XLH and its treatment	⨁⨁⨁⨁ High
B An orthopedic surgeon for timely surgical correction of skeletal deformities	⨁⨁⨁⨁ High
C A dentist or endodontist for regular screening, prevention, and timely treatment of dental conditions such as periodontitis or dental abscess	⨁⨁⨁⨁ High
D A neurosurgeon or craniofacial surgeon, in case of complications such as craniosynostosis, syringomyelia, or Chiari 1 malformation or abnormal clinical findings such as elevated intracranial pressure or headache	⨁⨁⨁⨁ High
E A physiotherapist and occupational therapist for improvement in muscle strength, stiffness, pain, mobility, gait, and optimal school participation, especially after orthopedic surgery	Expert opinion
F An otolaryngologist or audiologist for regular screening of hearing impairment	⨁⨁⨁◯ Moderate
Optimal multidisciplinary care of children with XLH may also include referral to the following specialties based on the available resources:	
G A clinical psychologist (or a trained social worker in some APAC settings) for counseling and improvement in mental health and stigma associated with XLH	⨁⨁⨁◯ Moderate
H A dietitian or nutritionist for counseling on healthy dietary choices	Expert opinion
I Geneticist or genetic counselor at the time of diagnosis of XLH	Expert opinion

Statement 8	GRADE evidence quality
We recommend that, if resources are available, an adult diagnosed with XLH should be referred to and followed up by a multidisciplinary team composed of:	
A physician with experience or special interest in XLH—often an adult endocrinologist but in some settings a nephrologist or general internist (internal medicine specialist)—for the medical treatment of metabolic bone disease and other complications (eg, pain) associated with XLH and its treatment	⨁⨁⨁⨁ High
B A physiotherapist, occupational therapist, physiatrist, or rehabilitation specialist for improvement in muscle strength, stiffness, pain, mobility, and workforce participation, especially after orthopedic surgery	⨁⨁⨁⨁ High
C A dentist or endodontist for screening, prevention, and timely treatment of dental conditions such as periodontitis or dental abscess	⨁⨁⨁⨁ High
D An orthopedic surgeon or rheumatologist, in case of any skeletal deformities or orthopedic conditions such as osteoarthritis or enthesopathies for timely correction and management of the conditions	⨁⨁⨁⨁ High
E Geneticist or genetic counselor, when planning for family	⨁⨁⨁◯ Moderate
Optimal multidisciplinary care of adults with XLH may also include referral to the following specialties, based on the clinical symptoms, available resources, and individual needs:	
F A neurosurgeon in case of neurologic complications such as Chiari 1 malformations, abnormal clinical findings such as elevated intracranial pressure or headache, or spinal stenosis	⨁⨁⨁◯ Moderate
G An audiologist or otolaryngologist for screening, early detection, and treatment of hearing impairment	⨁⨁⨁◯ Moderate
H A clinical psychologist for counseling and improvement in mental health and stigma associated with XLH	⨁⨁⨁◯ Moderate
I A gynecologist and obstetrician in case of women with XLH	Expert opinion
J A dietitian or nutritionist for counseling on healthy dietary choices	Expert opinion

Abbreviation: APAC = Asia‐Pacific; GRADE = Grading of Recommendations, Assessment, Development, and Evaluations; XLH = X‐linked hypophosphatemia.

**Table 5:** Asia‐Pacific Consensus Recommendations for the Follow‐Up Monitoring of XLH in Children

Monitoring assessment		GRADE evidence rating	Assessment frequency (expert opinion)
Statement 9: The recommended follow‐up monitoring assessments and their frequency in children with XLH to ensure optimized multidisciplinary treatment and care include:			
Clinical	Height/length, weight, BMI, head circumference, growth velocity, blood pressure	⨁⨁⨁⨁ High	1–3 months for at least 1 year after XLH diagnosis and treatment initiation, followed by 3‐monthly, thereafter. Blood pressure assessment should be initiated once the child is compliant with testing
	Limb pain (bone, muscle joint), headache		
	Intermalleolar distance, intercondylar distance, skull shape		
Biochemical	Serum calcium, serum or plasma phosphate, creatinine, ALP	⨁⨁⨁⨁ High	1–3 months for at least 1 year after XLH diagnosis and treatment initiation, followed by 3‐monthly, thereafter (creatinine and blood urea nitrogen to be monitored as needed, eg, in case of nephrocalcinosis)
	PTH	⨁⨁⨁⨁ High	
	Urine calcium: creatinine ratio	⨁⨁⨁⨁ High	Annually
	25‐hydroxy vitamin D	⨁⨁⨁⨁ High	Annually
	1,25‐dihydroxy vitamin D	⨁⨁⨁⨁ High	Annually, only in patients on burosumab, based on availability of resources
Radiological	Bone age X‐ray	⨁⨁⨁⨁ High	Once in every 1–2 years or as clinically indicated
	Other X‐ray imaging	⨁⨁⨁⨁ High	As clinically indicated
	Renal ultrasound	⨁⨁⨁⨁ High	Annually
Orthopedic		⨁⨁⨁⨁ High	Children with limb deformities, unexplained gait abnormalities, or persistent bone pain
Craniofacial		⨁⨁⨁◯ Moderate	Referral to neurosurgeon if resources are available: (1) For screening of complications such as craniosynostosis, syringomyelia, or Chiari 1 malformation; and (2) for treatment and subsequent monitoring in case of abnormal clinical findings such as elevated intracranial pressure, headache, etc.
Neurosurgical		⨁⨁⨁◯ Moderate	
Dental		⨁⨁⨁⨁ High	Twice yearly
Hearing		⨁⨁◯◯ Low	Assessment should be initiated at 8 years of age or earlier, as needed. Patients with hearing difficulties should be referred to an otolaryngologist for treatment and subsequent monitoring
Genetic counseling		Expert opinion	Counseling to parents and caregivers of children diagnosed with XLH at the time of diagnosis or for family planning
Functional	Developmental milestones, school participation	Expert opinion	At every clinic visit
	6MWT, ABC scale, sit‐to‐stand	⨁⨁⨁⨁ High	Annually, based on availability of resources
Psychosocial (using specific questionnaires)		⨁⨁⨁◯ Moderate	Annually, based on availability of resources

Abbreviation: ABC = activity‐specific balance confidence; 6MWT = 6‐minute walk test; ALP = alkaline phosphatase; BMI = body mass index; GRADE = Grading of Recommendations, Assessment, Development, and Evaluations; PTH = parathyroid hormone; XLH = X‐linked hypophosphatemia.

**Table 6:** Asia‐Pacific Consensus Recommendations for the Follow‐Up Monitoring of XLH in Adolescents

Monitoring assessment		GRADE evidence rating	Assessment frequency (expert opinion)
Statement 10: The recommended follow‐up monitoring assessments and their frequency in adolescents with XLH to ensure optimized multidisciplinary treatment and care include:			
Clinical	Height/length, growth velocity, weight, BMI, blood pressure	⨁⨁⨁◯ Moderate	1–3 months for at least 1 year after XLH diagnosis and treatment initiation, followed by 3‐monthly, thereafter
	Limb pain (bone, muscle joint, joint mobility), headache		
	Intermalleolar distance, intercondylar distance		
Biochemical	Serum calcium, serum/plasma phosphate, creatinine, ALP	⨁⨁⨁◯ Moderate	Every 3 months
	PTH	⨁⨁◯◯ Low	
	Urine calcium/creatinine ratio	⨁◯◯◯ Very low	Annually
	25‐hydroxy vitamin D	⨁◯◯◯ Very low	Annually
	1,25‐dihydroxy vitamin D	⨁◯◯◯ Very low	Annually, only in patients on burosumab based on availability of resources
Radiological	Bone age X‐ray	⨁⨁⨁⨁ High	Once every 1–2 years or as clinically indicated
	Other X‐ray imaging	⨁⨁⨁◯ Moderate	As clinically indicated
	Renal ultrasound	⨁⨁⨁◯ Moderate	Annually
Orthopedic		⨁⨁⨁⨁ High	Adolescents with limb deformities, unexplained gait abnormalities or persistent bone pain
Craniofacial		Expert opinion	Adolescents with relevant abnormal clinical findings suggesting elevated intracranial pressure, headache, etc., should be referred to neurosurgeon for treatment and subsequent monitoring
Neurosurgical		⨁⨁◯◯ Low	
Dental		⨁⨁⨁⨁ High	Twice yearly
Hearing		⨁◯◯◯ Very low	Adolescents with hearing difficulties should be referred to an otolaryngologist for treatment and subsequent monitoring
Genetic counseling		Expert opinion	During transition to adolescence
Functional	School participation, academic performance	Expert opinion	At every clinic visit
	6MWT, ABC scale, sit‐to‐stand	⨁⨁◯◯ Low	Annually, based on availability of resources
Psychosocial (using specific questionnaires)		⨁⨁⨁◯ Moderate	Annually, based on availability of resources

Abbreviation: ABC = activity‐specific balance confidence; 6MWT = 6‐minute walk test; ALP = alkaline phosphatase; BMI = body mass index; GRADE = Grading of Recommendations, Assessment, Development, and Evaluations; PTH = parathyroid hormone; XLH = X‐linked hypophosphatemia.

**Table 7:** Asia‐Pacific Consensus Recommendations for the Follow‐Up Monitoring of XLH in Adults

Monitoring assessment		GRADE evidence rating	Assessment frequency (expert opinion)
Statement 11: The recommended follow‐up monitoring assessments and their frequency in adults with XLH to ensure optimized multidisciplinary treatment and care include:			
Clinical	Height, weight, BMI, blood pressure	⨁⨁⨁⨁ High	Annually or at each follow‐up visit
	Limb pain (bone, muscle joint, joint mobility), headache		
Biochemical	Serum/plasma phosphate	⨁⨁⨁⨁ High	Every 3 months or at each follow‐up visit in symptomatic adults and/or adults undergoing treatment
	Serum calcium, serum creatinine, ALP	⨁⨁⨁⨁ High	Annually
	PTH	⨁⨁⨁⨁ High	Annually (every 3 months in patients with elevated PTH)
	25‐hydroxy vitamin D	⨁⨁⨁⨁ High	Annually
	1,25‐dihydroxy vitamin D	⨁◯◯◯ Very low	Annually, only in patients on burosumab based on availability of resources
Radiological	X‐ray imaging	⨁⨁⨁⨁ High	As indicated
	DXA	⨁⨁⨁◯ Moderate	As indicated (may be considered only in XLH patients aged >50 years or in postmenopausal women)
	Renal ultrasound	⨁⨁⨁⨁ High	Annually or every 2 years only in patients on conventional or burosumab therapy, or in patients with preexisting nephrocalcinosis and/or tertiary hyperparathyroidism
Orthopedic		⨁⨁⨁⨁ High	Adults with limb deformities, limb pain or fracture/pseudofractures should be referred to orthopedic surgeon for treatment and subsequent monitoring
Craniofacial		⨁⨁◯◯ Low	Adults with complications such as Chiari 1 malformations or spinal stenosis or relevant abnormal clinical findings such as elevated intracranial pressure, headache, etc., should be referred to neurosurgeon for treatment and subsequent monitoring
Neurosurgical		⨁⨁⨁◯ Moderate	
Dental		⨁⨁⨁⨁ High	Twice yearly
Hearing		⨁⨁◯◯ Low	Adults with hearing difficulties should be referred to an otolaryngologist for treatment and subsequent monitoring
Genetic counseling		Expert opinion	For women with XLH planning pregnancy or are pregnant along with their partners, and for women planning pregnancy or are pregnant with their XLH partners
Functional	6MWT, ABC, sit‐to‐stand, grip strength test	⨁⨁⨁⨁ High	Annually or every 2 years; regular assessment postsurgery, including one scheduled at 12 months
Psychosocial (using specific questionnaires)		⨁⨁⨁◯ Moderate	Annually, based on availability of resources

Abbreviation: ABC = activity‐specific balance confidence; 6MWT = 6‐minute walk test; ALP = alkaline phosphatase; BMI = body mass index; DXA = dual‐energy X‐ray absorptiometry; GRADE = Grading of Recommendations, Assessment, Development, and Evaluations; PTH = parathyroid hormone; XLH = X‐linked hypophosphatemia.

**Table 8:** Asia‐Pacific Consensus Recommendations on the Role of Telemedicine in Care for XLH

Statement 12	GRADE evidence quality: ⨁⨁⨁◯ Moderate
Telemedicine and digital medicine (in APAC settings, where it is legally approved) may help in supporting XLH patients to access care easily (eg, rural or remote settings) or when hospital admissions are restricted. However, telemedicine cannot replace face‐to‐face consultations, especially if physical examination and diagnostic imaging are required.	

Abbreviation: APAC = Asia‐Pacific; GRADE = Grading of Recommendations, Assessment, Development, and Evaluations; XLH = X‐linked hypophosphatemia.

The primary management goal in children with XLH is to promote optimal growth and global development, early correction of deformities, and monitoring for XLH complications and its treatment. When children with XLH enter adolescence, there is an added need to ensure that they are prepared for transition into the adult world, both medical and the wider community. Throughout childhood and adolescence, it is essential that the young person with XLH is supported at school and has full access to the educational curriculum.

In adult life, issues such as reproductive options and obstetric care should be addressed as a part of holistic care. Later, complications such as fractures, arthritis, and dystrophic calcification need to be addressed. Support needs to be provided in the workplace to ensure that the adult with XLH is able to achieve and maintain gainful employment. Across all ages, the overarching principles of care include early multidisciplinary care, early initiation and adherence to treatment, and early detection of complications. Psychosocial and emotional well‐being should also be considered at all stages of development in efforts to improve overall QoL.^(^ ^13^, ^49^, ^77^, ^78^, ^79^, ^80^ ^)^

#### Pharmacologic treatment

Table 3 (Statements 5A–5E and 6) summarizes our recommendations for the treatment of XLH in the APAC region.

##### Conventional therapy

The conventional medical therapy for XLH is oral phosphate supplementation, combined with active vitamin D (ie, calcitriol or alfacacidol) (Table 3, Statement 5A). Real‐world studies among children and adults have demonstrated the association of conventional therapy with improved outcomes in XLH, such as: (i) clinical outcomes (higher height *Z*‐scores), (ii) radiographic outcomes (fewer skeletal events), and (iii) dental health outcomes (lower carious index, lower attachment loss, fewer endodontically treated or absent teeth).^(^ ^32^, ^36^, ^37^, ^81^, ^82^, ^83^ ^)^

Among children with XLH, early initiation of conventional therapy has been associated with improved growth parameters. A retrospective cohort study in the UK (*N* = 23) showed that the most recent height measurements were significantly better among children who started conventional therapy before 1 year of age compared with those who started later (median height standard deviation score −0.7 versus −2.0, *p* = 0.009).^(^ ^47^ ^)^

Accordingly, international and national guidelines recommend starting combination therapy with oral phosphate and active vitamin D in children as soon as the diagnosis of XLH is established. The initial goal involves healing of rickets in affected children. Treatment is also aimed at reducing skeletal deformities, precluding surgery, facilitating growth, alleviating bone pain, and improving dental health. The generally recommended starting doses in children are 20–60 mg/kg body weight of elemental phosphorus given 4–6 times daily, and 20–40 ng/kg body weight of calcitriol given in two doses per day or 30–50 ng/kg body weight of alfacacidol given once daily.^(^ ^12^, ^13^, ^19^, ^22^, ^23^, ^84^ ^)^

Conventional therapy with oral phosphate and 1,25‐dihydroxy vitamin D_3_ in a prospective study in 16 symptomatic adults with XLH has been found to be associated with a significant increase in serum phosphate and reduction in osteoid thickness and osteoid volume. The reduction in osteoid volume correlated with a significant reduction in symptomatic bone/joint pain.^(^ ^85^ ^)^ In another prospective, observational study in 34 adults with XLH, continual treatment of hypophosphatemia with vitamin D and phosphate was associated with improvement in periodontal health.^(^ ^81^ ^)^ Apart from skeletal and oral health outcomes, conventional therapy in adults has been associated with improvements in mental health outcomes, such as better mental component scores in the 36‐item Short Form survey (SF‐36).^(^ ^14^, ^79^ ^)^ However, the evidence of clinical benefit is insufficient to support conventional treatment in asymptomatic adults.^(^ ^52^, ^79^ ^)^ Hence, contemporary guidelines recommend initiating or continuing combination therapy with oral phosphate and active vitamin D only in symptomatic adults, specifically those with dental problems, musculoskeletal pain, pseudofractures, elevated ALP, or planned orthopedic or dental surgery.^(^ ^12^, ^13^, ^22^ ^)^ In general, the recommended doses of conventional treatment for adults are 750–2000 mg of elemental phosphorus divided into 2–4 doses daily, and 0.50–0.75 μg of calcitriol or 0.75–1.5 μg of alfacacidol daily.^(^ ^12^, ^13^, ^22^, ^23^ ^)^

Despite the clinical benefits of conventional treatment, the multiple daily doses required may disrupt routine activities and become burdensome to patients. Along with the medications' unpleasant taste and potential for adverse effects, these factors may limit adherence to conventional therapy (Table 3, Statement 5B).^(^ ^16^, ^19^, ^86^ ^)^ Key adverse events include: (i) gastrointestinal discomfort (eg, diarrhea, bloating) in 56% of patients, (ii) hyperparathyroidism in 17%–47.5% of children and 15.6%–45% of adults, and (iii) nephrocalcinosis in 15.6%–25% of adults and 11.3%–68% of children.^(^ ^14^, ^15^, ^16^, ^35^, ^37^, ^39^, ^40^, ^43^, ^47^ ^)^ Nephrocalcinosis results from the promotion of hypercalciuria by conventional therapy and has been associated with longer treatment durations and higher treatment dosages.^(^ ^37^, ^39^ ^)^ Additionally, presence of nephrolithiasis has been documented in 2%–14% of XLH patients,^(^ ^16^, ^37^ ^)^ and impairment of renal function in 7.1% of children^(^ ^15^ ^)^ and 8%–9.5% of adults with XLH.^(^ ^15^, ^16^ ^)^ Furthermore, hypertension has been reported in treated XLH children with a significant association with hyperparathyroidism, which is one of the key side effects of conventional therapy.^(^ ^87^ ^)^

Commercial availability of oral phosphate solutions in the APAC region is limited. In many APAC countries, oral phosphate solutions need to be prepared in private pharmacies. Therefore, accessibility is curtailed, especially in rural or remote areas. Furthermore, oral phosphate supplementation and/or active vitamin D are not reimbursed under select national health insurance (NHI) schemes in some APAC regions. An impetus, therefore, exists for national governments and health agencies to ensure access to standard conventional therapy formulations for all children with XLH and all symptomatic adults with XLH in the APAC region (Table 3, Statement 5C).

Notwithstanding, the health‐related quality of life (HRQoL) of XLH patients remains poor even with conventional treatment, indicating the need for better treatment options.^(^ ^88^ ^)^

##### Burosumab

Burosumab is a fully human monoclonal antibody against FGF23. In 2018, the European Medicines Agency (EMA) approved its use in EU for XLH in children from the age of 1 year and adolescents with a growing skeleton. In the same year, the Food and Drug Administration granted approval of its use in children aged 1 year and older and subsequently also granted approval for patients 6 months of age and older. The EU approval was expanded further in 2020 to include all adolescents and adults.^(^ ^13^, ^89^, ^90^ ^)^

The efficacy of burosumab as treatment for pediatric XLH has been demonstrated in an international phase 3 RCT among children aged 1–12 years (*N* = 61). The study revealed the superiority of burosumab over conventional therapy at 64 weeks in terms of: (i) a greater increase in height (mean difference 0.14, *p* < 0.05); (ii) a greater decrease in rickets severity by radiographic impression of change score (mean difference 1.0, *p* < 0.0001) and by rickets severity score (RSS; mean difference −1.2, *p* < 0.0001); and (iii) a greater decrease in lower limb deformity (mean difference 1.0, *p* < 0.0001). Significant improvements in biochemical parameters (ie, serum phosphorus, TmP/GFR, ALP), pain interference scores, and 6MWT scores have also been reported. According to a post hoc subgroup analysis of this trial, the improvements with burosumab versus conventional therapy, specifically in terms of the biochemical parameters, are found both in children aged 1–<5 years and those aged 5–12 years.^(^ ^48^, ^57^, ^91^ ^)^

Most contemporary guidelines recommend initiation of burosumab in patients who have (i) rickets that is radiographically proven and refractory to conventional therapy or (ii) intolerance or complications with conventional therapy. The recommended starting dose of burosumab in children is 0.8 mg/kg body weight given subcutaneously every 2 weeks. Conventional therapy must be discontinued 2 weeks before burosumab therapy, and the child should have a serum phosphate level below the lower end of the age‐adjusted reference range.^(^ ^12^, ^13^, ^19^, ^21^, ^22^, ^23^ ^)^

Among adults, the clinical benefits of burosumab therapy over placebo have also been illustrated by RCTs.^(^ ^50^, ^51^, ^67^, ^92^ ^)^ In a large‐scale, international, phase 3 RCT among adults with XLH (*N* = 134), the burosumab group had a significantly higher proportion of subjects achieving a normalization in serum phosphate versus the placebo group (94.1% versus 7.6%, *p* < 0.001) at 24 weeks, as well as greater improvements in fracture/pseudofracture healing, other biochemical parameters, and patient‐reported outcomes (PROs).^(^ ^50^ ^)^ An extension of this study revealed persistent improvements with burosumab beyond 24 weeks, particularly in terms of PROs (pain, stiffness, fatigue) and functional capacity.^(^ ^51^, ^67^ ^)^ However, available evidence of efficacy versus conventional therapy is limited, and hence, current guidelines recommend burosumab therapy for adults with refractory symptoms or those experiencing complications with conventional therapy.^(^ ^12^, ^13^, ^22^, ^23^ ^)^ In adults, the recommended initial dose of burosumab is 1.0 mg/kg body weight given subcutaneously every 4 weeks.^(^ ^12^, ^22^, ^23^ ^)^ As with children, conventional therapy should not be used concurrently with burosumab therapy.^(^ ^12^, ^22^ ^)^ Our recommendations for burosumab therapy in the APAC region can be found in Table 3, Statement 5D.

In contrast to conventional therapy, burosumab is not associated with adverse effects such as nephrocalcinosis and hyperparathyroidism.^(^ ^93^, ^94^ ^)^ In clinical trials of burosumab therapy, nephrocalcinosis did not develop among previously unaffected patients, whereas substantial progression was not detected in nearly all of those with baseline affectation.^(^ ^50^, ^57^, ^58^, ^59^, ^63^, ^95^, ^96^, ^97^ ^)^ Nevertheless, adverse events with at least a possible relation to burosumab have been reported in 38.5%–59% of children and 64%–71.4% of adults in clinical trials.^(^ ^57^, ^59^, ^63^, ^95^ ^)^ The most common adverse effect was injection site reaction (eg, erythema, urticaria), which was mostly mild, limited to the skin, and resolved in a few days.^(^ ^50^, ^57^, ^58^, ^59^, ^63^, ^95^ ^)^ Based on follow‐up studies of RCTs, the safety and tolerability of burosumab are sustained through ~120–160 weeks of treatment.^(^ ^96^, ^97^ ^)^

Apart from a small‐scale phase 3/4, single‐arm trial in Japan (*N* = 15),^(^ ^97^ ^)^ the studies evaluating burosumab therapy have involved predominantly Western populations. In a few international RCTs, Asian subjects have comprised only ~13%–15.7% of study participants.^(^ ^48^, ^50^, ^51^, ^57^, ^67^ ^)^ More research conducted in the APAC region is warranted to formulate more specific guidelines on burosumab therapy directed toward APAC patients. Currently, burosumab is not consistently available across the APAC region (Table 3, Statement 5E). In APAC countries where burosumab is available, accessibility is restricted by high costs. If approved, coverage under the NHI scheme of APAC countries is expected to increase access of XLH patients to burosumab therapy. For instance, burosumab has recently been added into the Pharmaceutical Benefits Scheme of Australia, whereby the government would subsidize its costs for both children and adults with XLH.^(^ ^98^ ^)^

##### Growth hormone

Current guidelines do not recommend treatment of XLH children with short stature with recombinant human growth hormone (rhGH)^(^ ^23^ ^)^ due to the lack of high‐quality evidence supporting its use.^(^ ^99^ ^)^ However, in a recent retrospective longitudinal cohort study, 2 years of rhGH therapy was associated with improvement in final height in 34 XLH children with growth failure despite conventional treatment.^(^ ^100^ ^)^ In another prospective, longitudinal, observational cohort study, addition of rhGH to conventional or burosumab therapy was noted to be safe and resulted in catch‐up growth in 13 children with XLH.^(^ ^101^ ^)^ Because of the low sample size and observational nature of these studies, no clear recommendation can be made for the use of growth hormone in children with XLH and short stature. Well‐designed RCTs in the future may help establish the role of growth hormone for the treatment of children with XLH.

##### Pregnancy and lactation

Published literature surrounding XLH in pregnancy is sparse (Table 3, Statement 6). In a report from a single center, conventional therapy was continued among stable pregnant women with XLH, although it was not initiated in those not previously undergoing treatment. Overall, there was a small increase in urine calcium/creatinine ratio in patients on conventional treatment; however, there was no significant association of this finding with nephrocalcinosis. All pregnant women with XLH on conventional therapy were monitored for serum calcium and urinary calcium/creatinine ratio, with required treatment modifications. The rate of caesarean section was high (75%), primarily because of breech presentation and the clinician's/patient's preference owing to the potential complications associated with delivery in this patient cohort.^(^ ^102^ ^)^

No prior consensus or strong recommendation has been established regarding conventional therapy for pregnant or lactating patients with XLH.^(^ ^12^, ^13^, ^22^, ^23^ ^)^ Nevertheless, pregnancy and lactation are critical periods for bone health, as bone mineralization disorders may also exacerbate during pregnancy.^(^ ^103^ ^)^ Therefore, intensification of monitoring under these circumstances is essential. More studies in the future may help in optimizing the guidance on conventional treatment and monitoring of pregnant and lactating women with XLH.

There are no or limited data on the use of burosumab in pregnancy.^(^ ^89^, ^90^ ^)^ Similarly, no information is available regarding presence of burosumab in breastmilk or its effects on the breastfed infant.^(^ ^89^, ^90^ ^)^ The EMA labeling of burosumab does not recommend its use during pregnancy and in women of childbearing potential not using contraception.^(^ ^89^ ^)^ The post‐authorization safety study (PASS) is a retrospective and prospective cohort study that will utilize the data from the international XLH registry to evaluate the frequency and outcomes of pregnancies in female XLH patients receiving burosumab.^(^ ^104^ ^)^

#### Multidisciplinary referral

The recommended multidisciplinary referral for XLH patients is shown in Table 4 (Statements 7 and 8). The multidisciplinary team managing XLH cases is frequently led by a metabolic bone specialist or an endocrinologist (pediatric or adult). In some settings, the team may be steered by a pediatrician or internist with special interest in XLH or a nephrologist (pediatric or adult).^(^ ^53^ ^)^ In any case, the lead physician should facilitate the development of a multidisciplinary management team including at least orthopedics, dentistry, rehabilitation medicine, and allied health for both children and adults with XLH.

About 50%–80% of children and adults with XLH will undergo at least one orthopedic procedure, warranting the presence of an orthopedic surgeon in the multidisciplinary team.^(^ ^16^, ^37^, ^43^, ^53^ ^)^ Among children, surgeries commonly performed include hemi‐epiphysiodesis (14.3%–19%) during the growth phase and osteotomy (17%–33%) when growth has ceased.^(^ ^15^, ^16^, ^39^ ^)^ Expectedly, more frequent osteotomies (34.4%–65.5%) and fewer hemi‐epiphysiodesis (6%) are found in adults.^(^ ^15^, ^16^, ^82^ ^)^ Adults may also need to undergo surgical repair of fractures.^(^ ^16^, ^102^ ^)^ Moreover, because osteoarthritis is prevalent in adult XLH, surgical procedures also include knee and hip replacements.^(^ ^14^, ^16^, ^102^ ^)^ For similar reasons, referral to a rheumatologist may be necessary.

The propensity of those with XLH for developing oral health issues requires the involvement of dental services in the management team. Endodontic treatment may be necessary in both children and adults;^(^ ^15^, ^16^, ^36^, ^81^ ^)^ a history of root canal surgery is found in up to 72% of adults with XLH.^(^ ^16^ ^)^ Meanwhile, dental extraction is performed in about one‐third of children and half of adolescents and adults with XLH.^(^ ^63^, ^102^ ^)^

Rehabilitation in XLH, which aims to improve daily function, may involve physiotherapists, occupational therapists, physiatrists, or rehabilitation medicine specialists. Assistive devices for mobility (eg, wheelchair, walking device) are used in both pediatric and adult XLH patients with varying prevalence (11.5%–100%).^(^ ^14^, ^15^, ^37^, ^105^ ^)^ Additionally, 10.2%–57.4% of XLH patients undergo physical therapy or physiotherapy.

Because of comorbidities such as Chiari malformation, craniosynostosis, and syringomyelia, 3.3%–9% of children and 2.6%–6.3% of adults would need neurosurgery (eg, craniotomy, craniectomy) warranting referral to a neurosurgeon.^(^ ^15^, ^16^, ^102^, ^106^ ^)^ Moreover, hearing impairment or loss may be found in 5.7%–55% of children and adults with XLH, necessitating a referral for formal audiologic assessment starting at 8 years of age.^(^ ^12^, ^15^, ^16^, ^53^, ^102^ ^)^ Contemporary guidelines also recommend referrals to geneticists and genetic counselors for genetic counseling, psychologists for psychosocial support, rehabilitation specialists for supporting access to educational curriculum and workforce participation, and dietitians for nutritional advice.^(^ ^12^, ^13^, ^19^, ^21^, ^22^, ^23^ ^)^ Among patients planning for pregnancy, a referral to a gynecologist or obstetrician should be made in addition to a genetics consult.

#### Monitoring and follow‐up

Monitoring and follow‐up is an important aspect of XLH management and should reflect the goals of multidisciplinary management at each life stage: childhood (Table 5, Statement 9), adolescence (Table 6, Statement 10), and adulthood (Table 7, Statement 11).

The frequency and interval of follow‐up should be individualized. However, in general, children and adolescents should be seen every 3 months, especially during infancy and puberty, wherein growth is most rapid. Adults require less frequent visits (every 6–12 months). Our recommendations on follow‐up evaluations and testing frequencies may vary from the international guideline recommendations and reflect the minimum standard, accounting for resource limitations in some APAC countries.

##### Clinical evaluation

Anthropometric measurements, such as height or length and weight, are essential components of each visit at any age. A higher body mass index (BMI) in XLH has been associated with gait deviations as well as higher counts of enthesopathies.^(^ ^78^, ^82^ ^)^ Patients with XLH also potentially have a higher prevalence of obesity versus the general population.^(^ ^17^ ^)^ In children, height *Z*‐scores and growth velocity should be determined, having demonstrated response to or correlation with treatment in previous literature.^(^ ^57^, ^83^ ^)^

Head circumference, skull shape, and neurologic symptoms (eg, headache) should be assessed at each visit during childhood to ensure that craniosynostosis or Chiari malformation are detected as early as possible.^(^ ^106^ ^)^ Regardless of age, limb deformities should be monitored during each visit, and intermalleolar and intercondylar distance must be measured.^(^ ^107^ ^)^ Additionally, the blood pressure should be monitored, in line with some evidence pointing to a higher prevalence and early onset of hypertension in XLH versus the general population, while on conventional medical therapy.^(^ ^87^, ^108^ ^)^

Pain and joint stiffness are symptoms known to respond to treatment and should be elicited during consultation. Although previous studies involving children have used the Patient‐Reported Outcomes Measurement Information System (pain interference, physical function mobility, fatigue),^(^ ^48^ ^)^ studies involving adults have applied a variety of PROs validated for XLH, including the Brief Pain Inventory Short Form (pain severity and interference) and Western Ontario and McMaster Universities Osteoarthritis Index (WOMAC; pain, stiffness, physical function).^(^ ^50^, ^51^, ^67^ ^)^ PROs such as the WOMAC pain and stiffness score also correlate with counts of enthesopathies and presence of fractures.^(^ ^37^ ^)^

For patients aged ≥5 years, physical function may be evaluated using parameters of the 6MWT (eg, total distance, percentage of predicted distance), which have shown improvement with treatment in prior studies.^(^ ^51^, ^57^, ^67^ ^)^ The Activities‐Specific Balance Confidence (ABC) scale may also be utilized, wherein lower scores correlate with fear of falling, while higher scores correlate with greater range of motion.^(^ ^105^ ^)^ Because of potential muscle‐bone cross‐talk,^(^ ^109^ ^)^ monitoring muscle function via handgrip strength or the sit‐to‐stand test may also be useful and considered as per the local resource constraints.

Other questionnaires for health‐related quality of life (SF‐36, Health Assessment Questionnaire, Routine Assessment of Patient Index 3) have not been validated in XLH but have illustrated treatment response, as well as correlation with deformities, dental health, and enthesopathies in past studies.^(^ ^79^ ^)^

##### Biochemical evaluation

Biochemical assessments are performed regularly in XLH to monitor disease activity, adjust treatment, and balance therapeutic response and safety. Accordingly, the frequency of testing is higher among patients who are symptomatic or on pharmacologic therapy (ie, children) and lower among asymptomatic patients not receiving treatment (ie, adults).

Total ALP in children and bone‐specific ALP in adults are markers of bone turnover and elevated with rickets and osteomalacia and should be determined regardless of treatment status or type of treatment. Although total ALP levels have improved among children treated with burosumab in clinical trials,^(^ ^57^ ^)^ bone‐specific ALP levels have been reported to increase from baseline after initiation of burosumab in adults, followed by gradual attenuation of this effect over time.^(^ ^50^, ^51^ ^)^ Among those receiving conventional therapy, ALP levels can be used to titrate treatment dosages.^(^ ^84^, ^86^, ^110^ ^)^ Most centers perceive ALP normalization as the primary biochemical parameter indicating satisfactory treatment.^(^ ^53^ ^)^ Although ALP levels ideally should be kept within the normal range, some believe levels below 1.5‐times the upper limit of normal for age are acceptable.^(^ ^53^ ^)^

Monitoring of serum phosphate is important during treatment of XLH. The goal of treatment with burosumab is to achieve phosphate homeostasis. Hence, serum phosphate levels should be monitored and used to adjust the dose of burosumab.^(^ ^50^, ^51^, ^57^, ^58^, ^63^, ^92^, ^95^ ^)^ On the other hand, conventional therapy will not restore phosphate levels and is not intended for normalizing phosphatemia.^(^ ^12^, ^13^, ^19^, ^23^ ^)^

Other biochemical tests conducted to detect potential adverse effects of pharmacologic therapy include blood levels of PTH and calcium; 25‐hydroxyvitamin D or 1,25‐dihydroxyvitamin D; urinary excretion of calcium (ie, urine calcium/creatinine ratio); and serum creatinine for calculation of GFR.^(^ ^35^, ^37^, ^40^, ^43^, ^47^, ^50^, ^51^, ^53^, ^57^, ^58^, ^59^, ^92^, ^95^, ^102^ ^)^ Phosphate supplementation may lead to hypocalcemia and stimulate parathyroid cells, causing secondary and eventually tertiary hyperparathyroidism,^(^ ^111^, ^112^ ^)^ although active vitamin D may potentially cause hypercalciuria and consequent nephrocalcinosis,^(^ ^113^ ^)^ with or without renal impairment. Burosumab therapy, which can increase blood levels of 1,25‐dihydroxyvitamin D, also has the potential to cause biochemical derangements downstream of this pathway.^(^ ^50^, ^51^, ^57^, ^58^, ^59^, ^92^, ^95^ ^)^

##### Radiologic evaluation

Serial X‐rays are recommended to monitor severity of rickets in children and adolescents with XLH. For instance, the rickets severity score based on wrist and knee radiography is not only known to improve with treatment but also correlates with height *Z*‐score, severity of dental abscesses, and PTH levels.^(^ ^37^, ^38^, ^57^ ^)^ These X‐rays can also be used to determine bone age and assess growth potential, especially among children with short stature.

To minimize radiation exposure, previous guidelines recommend that other X‐rays (eg, long bone radiography) be reserved for specific indications, including persistent lower limb deformities and need for orthopedic surgery (eg, for fractures).^(^ ^12^, ^13^, ^22^, ^23^ ^)^ Monitoring with X‐rays in clinical trials have shown that lower limb deformities and fractures/pseudofractures respond to burosumab therapy.^(^ ^50^, ^51^, ^57^ ^)^

Dual‐energy X‐ray absorptiometry (DXA) is a common diagnostic procedure to assess bone mineral density (BMD) and the associated risk for fractures among older adults and postmenopausal women with XLH.^(^ ^114^ ^)^ In children and adults with XLH, BMD is higher than healthy controls by DXA.^(^ ^115^, ^116^ ^)^ However, this finding is attributable to extraskeletal spinal calcifications and enthesopathies, exhibited by adults, who then require this imaging more than children.^(^ ^115^, ^116^ ^)^

Meanwhile, renal ultrasound has been the monitoring tool utilized in clinical trials and real‐world studies to evaluate the adverse effects of pharmacologic therapy in XLH.^(^ ^35^, ^37^, ^40^, ^47^, ^50^, ^51^, ^53^, ^57^, ^58^, ^59^, ^63^, ^92^, ^95^, ^102^ ^)^ Accordingly, it is the preferred screening method for nephrocalcinosis and should be performed annually.

##### Multidisciplinary monitoring

Because of the burden of dental disease in XLH, twice‐yearly dental evaluations are recommended, regardless of age.^(^ ^35^, ^38^, ^40^, ^43^, ^52^, ^53^, ^59^ ^)^ Monitoring of other complications (ie, orthopedic, craniofacial, neurosurgical, audiologic) should be individualized based on initial results, input from specialty referral, and availability of resources. Genetic counseling should be considered not only at the time of diagnosis but also during transition to adult care and during family planning. Moreover, regular psychosocial assessments should be performed in XLH patients across the life stages. Although these services may not be available in all clinical settings across the APAC region, the psychological health of children and adults with XLH must be considered and managed appropriately. In children and adolescents, checking for developmental milestones and performance in school should be a routine part of the health visit.^(^ ^18^, ^79^ ^)^

#### Role of telemedicine

A recommendation on the role of telemedicine in optimization of XLH treatment and care is provided in Table 8 (Statement 12). The COVID‐19 pandemic and corresponding public health interventions (eg, lockdown) caused a disruption of regular health services, including reduced hospital admissions and suspended outpatient operations. In Italy, telemedicine became an integral component of the management of XLH patients during the pandemic. Physicians communicated with patients and their caregivers through e‐mail, phone calls, or social media. Some clinical parameters (eg, anthropometrics, adverse events) required remote monitoring through photographs and videos. PROs (eg, WOMAC) were regularly administered as appropriate.^(^ ^117^ ^)^

In some APAC countries, even before the pandemic, certain areas may already be geographically disadvantaged, while others may lack centers of XLH expertise, and telemedicine may be essential for health care delivery in such countries/regions. Utilization of e‐health technologies has been reported to have advantages such as increased patient awareness of disease, better patient‐physician relationship, and reduced transportation and work burden. Although telemedicine may help reduce visits that do not need blood tests or imaging and may be useful in geographically challenged regions, it may not completely replace in‐person visits, especially when physical examination, imaging, blood tests, and multidisciplinary monitoring and referral are required in XLH.^(^ ^117^ ^)^

### Transition of care from childhood to adulthood

**Fig. 3:**
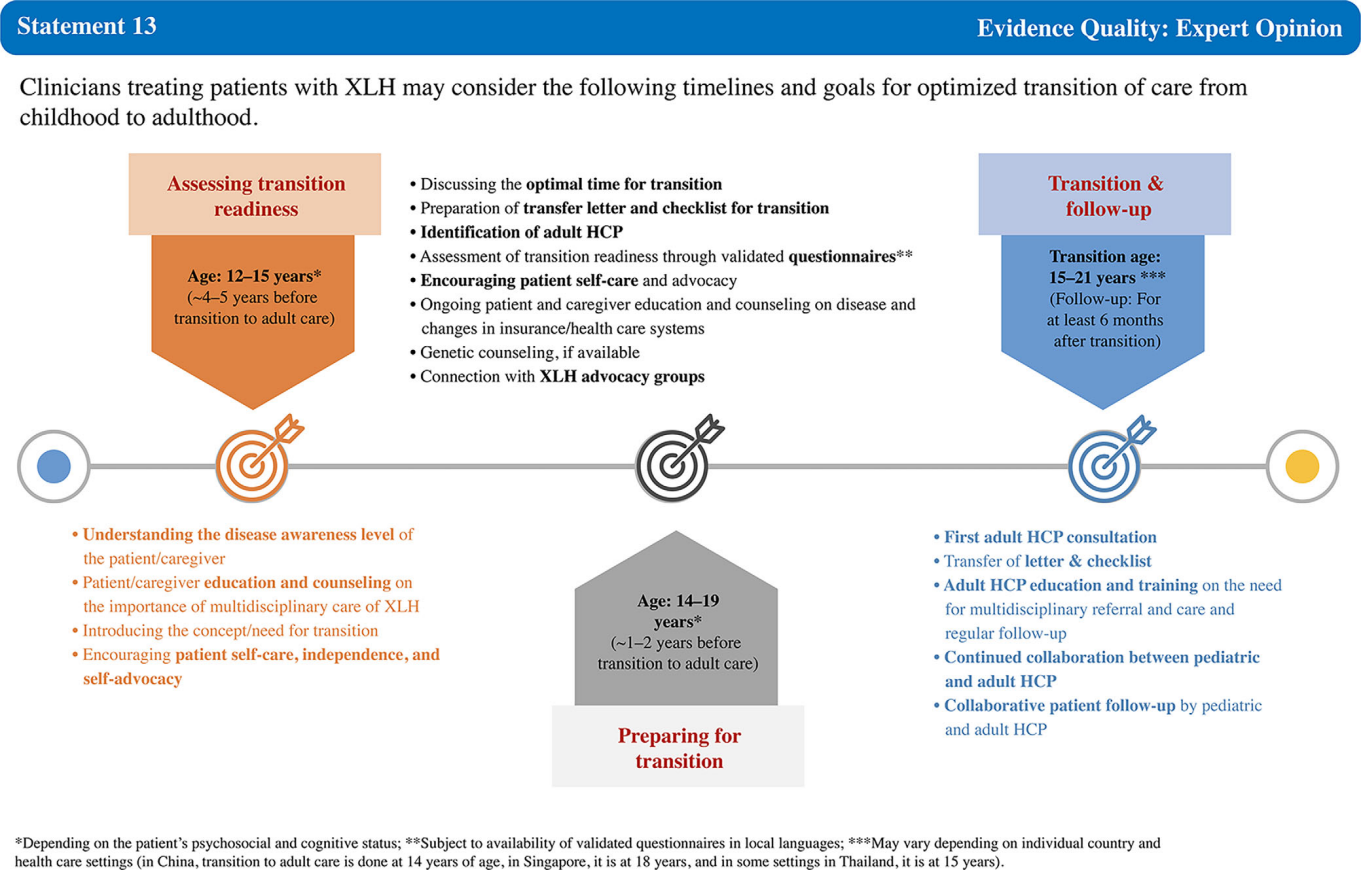
Asia‐Pacific consensus recommendations on timelines and goals for optimized transition of care of X‐linked hypophosphatemia (XLH). HCP = health care provider.

**Fig. 4:**
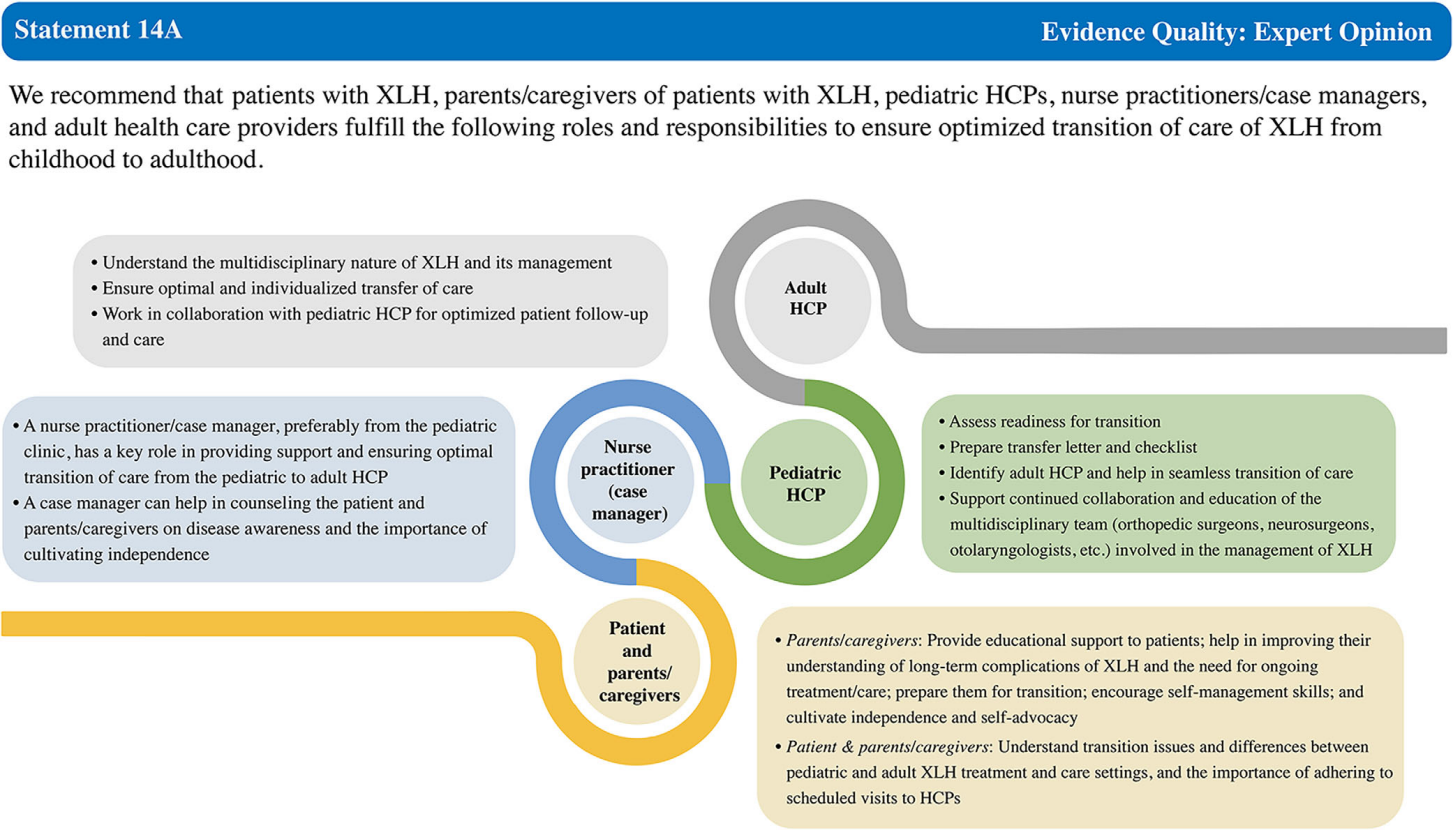
Asia‐Pacific consensus recommendations on roles and responsibilities to ensure optimized transition of care of X‐linked hypophosphatemia (XLH). HCP = health care provider.

Our recommendations for optimizing the transition of care of adolescents with XLH in the APAC region are shown in Fig. 3 (Statement 13), Fig. 4 (Statement 14A), Table 9 (Statements 14B and 14C), and Table 10 (Statement 15). All adolescents with XLH have the need for a carefully planned health care transition. In general, the health care transition can be divided into various stages: initiation and assessment of readiness for transition, proceeding with transition, and successful transition to adult care. Various models of care have been built for more common diseases, identifying the determinants of transition outcomes (eg, family and psychosocial support, effective communication). However, usual transition models may have restricted applicability to rare genetic diseases for several reasons: (i) limitations in access to a variety of specialists, (ii) the consequent lack of knowledge of disease, and (iii) the influence of long‐term caregiver experience on the expectations of the adolescent.^(^ ^20^ ^)^

**Table 9:** Asia‐Pacific Consensus Recommendations for the Use of Transition Questionnaires and Transition Clinics in XLH

Statement 14B	Evidence quality: Expert opinion
Validated transition questionnaires such as the TRAQ may be considered starting 2 years before transition, followed by every 1–2 years until transition, to assess the transition readiness of patients and parents/caregivers, in settings where it is feasible to use the validated English version of the questionnaire. Local validated (translated) versions of TRAQ may have to be developed to be used in specific local settings.	

Statement 14C	Evidence quality: Expert opinion
Transition clinics are important for optimizing the transition of care of XLH patients from childhood to adulthood. If resources are available, a typical “bone and metabolic disorder” or “XLH” transition clinic may be composed of a pediatric‐treating specialist, an adult‐treating specialist, a nurse or case manager, patients with XLH, and parents or caregivers. The first transition clinic may be conducted in a familiar setting such as the pediatric clinic to provide a supportive environment to the patient or at a “bone and metabolic disorder” clinic, if available, to help in seamless transition of care.	

Abbreviation: TRAQ = Transition Readiness Assessment Questionnaire; XLH = X‐linked hypophosphatemia.

**Table 10:** Asia‐Pacific Consensus Recommendations on the Checklist to be Passed From the Pediatric to Adult Health Care Provider During Transition of Care of XLH

Statement 15		Evidence quality: Expert opinion	
The checklist of information that should be passed along with the transfer letter from the pediatric to adult health care provider during the transition of care of patients with XLH from childhood to adulthood may include the following parameters:			
**Patient/general information**			
**Patient information**	**Details**	**General health care information**	**Details**
Name		Details of comorbid medical conditions (if any)	
Date of birth/current age		Current medications	
Date of transfer of care		Current health care provider (name and contact details)	
Sex		Previous health care providers (name and contact details)	
Address and contact number		New health care provider (name and contact details)	
Current insurance details		Details of prior participation in clinical trials	
Informed consent for transition	□ Yes □ No	Additional details (if any)	
Additional details (if any)			
**XLH history**			
**XLH disease history**	**Details**	**Documents related to disease history** (as attachments)	**Details**
Age at diagnosis		Renal ultrasound	
Lab results at diagnosis		Radiographs	
Clinical features at diagnosis		Additional imaging results (if any)	
Genetic test results (optional)		Dental images (optional)/records	
Lab results at latest visit		Gait video (optional)	
Hearing evaluation results		Detailed family history and photos (optional)	
Growth chart		Additional documents (if any)	
Functional outcomes (optional)			
Additional details (if any)			
**Treatment history (related to XLH)**			
**Medical treatment**	**Details of regimen/dose/duration**	**Surgical procedures**	**Details**
Phosphate salts		Hemi/epiphysiodesis	
Calcitriol		Osteotomy	
Alfacalcidol		Surgery for craniosynostosis	
Burosumab		Additional surgeries (if any)	
Other treatments (if any)			
**Complications**			
**XLH‐related complications**	**Details**	**Treatment‐related complications**	**Details**
Fractures		Nephrocalcinosis/nephrolithiasis	
Pseudofractures		Hyperparathyroidism	
Lower limb deformities		Hypercalciuria/hypercalcemia	
Dental conditions		Renal impairment	
Craniosynostosis		Additional adverse effects (if any)	
Chiari 1 malformation			
Additional complications (if any)			
**Rare disease/XLH advocacy groups/helplines**			
Advocacy groups or psychosocial support	□ Yes □ No	Helplines available	□ Yes □ No
If yes, details		If yes, details	
**Additional details (if any)**			

Abbreviation: XLH = X‐linked hypophosphatemia.

Although the evidence surrounding transition of care for XLH patients is lacking, overarching principles from transition models for other rare diseases remain indispensable in XLH. As with these other illnesses, timelines in XLH should be specified, consisting not only of well‐defined goals for each transition stage (Fig. 3, Statement 13) but also clear and distinct roles and responsibilities for all involved stakeholders (Fig. 4, Statement 14A). Patient education, geared toward increasing awareness, independence, and self‐advocacy, is an encompassing theme across the entire course of transition, as is the provision of continuous psychosocial support.

In the APAC region, the cut‐off age for transition of care has a wide range (14–18 years) across countries. Generally, introducing transition of care to families should begin 2–4 years before the actual initiation of care transition. Involving the child in these discussions should be considered as early as the psychosocial status of the patient permits. However, the age at which these talks of transition commence also differs among APAC countries, varying based on the culturally accepted age when children assert their independence from their parents. Notwithstanding, timelines should be individualized according to the patient's readiness for transition of care. Additionally, in some countries or settings, a provision should be made to allow the pediatric XLH expert continue the follow‐up of patients with XLH, while facilitating remote consultation with adult XLH experts, if feasible to ensure continuity of care.

The infrastructure that supports the age‐appropriate and seamless transition involves transition questionnaires and transition clinics (Table 9, Statements 14B and 14C), as well as transfer letter checklists (Table 10, Statement 15). Transition questionnaires such as the TRxANSITION Index and the STARx Questionnaires have been validated in XLH.^(^ ^20^ ^)^ However, translations of these questionnaires to APAC languages are limited.^(^ ^118^ ^)^ Meanwhile, the Transition Readiness Assessment Questionnaire (TRAQ) has been translated into more APAC languages but are not specific to XLH (Table 9, Statement 14B).^(^ ^119^ ^)^

Although establishing a transition clinic will optimize the transfer of care (Table 9, Statement 14C), this setup may not be feasible in resource‐limited APAC settings. Thus, an XLH transition clinic may alternatively be enveloped under a more general clinic for bone and metabolic disorders. These clinics may meet once to twice per year, depending on availability of resources. The minimum requirement for a transition clinic will be the simultaneous presence of a pediatric and adult endocrinologist. However, enlisting the help of a case manager (eg, nurse practitioner) is ideal to provide a consistent connection between the patient and the pediatric and adult providers, even beyond transition clinic days.

Lastly, a transfer checklist containing details of care (eg, disease‐specific information, physician data) facilitates a smoother transition between providers.^(^ ^20^ ^)^ Although such checklists should aim to be exhaustive, they should also be individualized and tailored to the APAC setting (Table 10, Statement 15).

### Medical education, training, and research

Continuing medical education and training for health care providers of XLH patients are a fundamental component of optimizing XLH care in the APAC region (Table 11, Statement 16). These initiatives are intended to address the limited number of multidisciplinary experts in APAC countries, as well as to extend the reach of their expertise toward remote areas with limited access to XLH care. Conducting focus group meetings among clinicians involved in the care of XLH patients, establishing “centers of excellence” across the APAC region, and utilizing telemedicine are potential means to achieve this goal. Additionally, establishing an APAC XLH registry in collaboration with key regional or local societies will help create a robust database to facilitate potential research in the future. Furthermore, generation of local data pertaining to response to treatment of XLH and complications will help in improving the utilization of newer treatment approaches such as burosumab and optimizing the management of XLH in the region.

**Table 11:** Asia‐Pacific Consensus Recommendations for Continuing Medical Education and Training in Care for XLH

Statement 16	Evidence quality: Expert opinion
The educational or training initiatives that may help pediatric and adult XLH experts to optimize the multidisciplinary management and transition of care of XLH, especially in settings with lack of access to pediatric and/or adult XLH experts include: Developing certified e‐learning courses for general practitioners, general internists (internal medicine specialists), endocrinologists, nephrologists, and pediatricians, in medical school/medical societies on: Diagnosis and management of XLH Transition of care of XLH from childhood to adulthood Establishing “XLH Centers of Excellence” with XLH experts who can: Provide online/teleconsultation to HCPs in regions with lack of XLH experts Offer preceptorship programs to train selected HCPs in the management and care of XLH patients Organizing focus group meetings, educational symposia, or training workshops in collaboration with local endocrine or rare disease societies Establishing APAC XLH registry in collaboration with key regional/local societies	

Abbreviation: APAC = Asia‐Pacific; GRADE = Grading of Recommendations, Assessment, Development, and Evaluations; HCP = health care provider; XLH = X‐linked hypophosphatemia.

## Conclusion

We conducted a comprehensive review of the existing literature on XLH and presented 16 consensus statements addressing the screening and diagnosis, multidisciplinary management, and transition of care of XLH from childhood to adulthood. Ultimately, optimized care for XLH patients requires prompt identification and early diagnosis; timely multidisciplinary care with regular monitoring and follow‐up; and a seamless transfer of care through the coordinated effort of all stakeholders. Based on the best available evidence, we outlined specific guidance for clinical practice in APAC settings. Nevertheless, more APAC research is warranted to refine future recommendations.

## Author Contributions

**Craig F Munns:** Conceptualization; data curation; methodology; project administration; supervision; validation; visualization; writing – original draft; writing – review and editing. **Han‐Wook Yoo:** Conceptualization; data curation; methodology; validation; visualization; writing – original draft; writing – review and editing. **Muhammad Yazid Jalaludin:** Conceptualization; data curation; methodology; validation; visualization; writing – original draft; writing – review and editing. **Rashida Vasanwala:** Conceptualization; data curation; methodology; validation; visualization; writing – original draft; writing – review and editing. **Manju Chandran:** Conceptualization; data curation; methodology; validation; visualization; writing – original draft; writing – review and editing. **Yumie Rhee:** Conceptualization; data curation; methodology; validation; visualization; writing – original draft; writing – review and editing. **Wai Man BUT:** Conceptualization; data curation; methodology; validation; visualization; writing – original draft; writing – review and editing. **Alice Pik‐Shan Kong:** Conceptualization; data curation; methodology; validation; visualization; writing – original draft; writing – review and editing. **Pen‐Hua Su:** Conceptualization; data curation; methodology; validation; visualization; writing – original draft; writing – review and editing. **Nawaporn Numbenjapon:** Conceptualization; data curation; methodology; validation; visualization; writing – original draft; writing – review and editing. **Noriyuki Namba:** Conceptualization; data curation; methodology; validation; visualization; writing – original draft; writing – review and editing. **Yasuo Imanishi:** Conceptualization; data curation; methodology; validation; visualization; writing – original draft; writing – review and editing. **Roderick J Clifton‐Bligh:** Conceptualization; data curation; methodology; validation; visualization; writing – original draft; writing – review and editing. **Xiaoping Luo:** Conceptualization; data curation; methodology; validation; visualization; writing – original draft; writing – review and editing. **Weibo Xia:** Conceptualization; data curation; methodology; project administration; supervision; validation; visualization; writing – original draft; writing – review and editing.

## Disclosures

CFM is a consultant for Kyowa Kirin and has received speaker fees, and research support from Kyowa Kirin. HWY and MYJ have received honoraria from Kyowa Kirin for speaking engagements. MC has received honoraria from Kyowa Kirin for speaking and chairing engagements. RFV, WMB, and PHS have received speaker and advisor fee from Kyowa Kirin. YR is an investigator in an ongoing, observational study conducted by Kyowa Kirin, and has received honoraria and research fund from Amgen. APK has received honoraria for consultancy and speaking engagements from Abbott, AstraZeneca, Bayer, Boehringer Ingelheim, Eli‐Lilly, Kyowa Kirin, Merck Serono, Nestle, Novo‐Nordisk, Pfizer and Sanofi. NN* has received speaker fees from Kyowa Kirin, Novo Nordisk and Ferring Pharmaceuticals. NN** has received speaker and advisor fees from Kyowa Kirin and is an investigator in an ongoing, observational study conducted by Kyowa Kirin. XL has no conflicts of interest to declare. YI, RCB, and WX have received research grants and consulting fees from Kyowa Kirin. *Nawaporn Numbenjapon; **Noriyuki Namba.

### Peer Review

The peer review history for this article is available at https://www.webofscience.com/api/gateway/wos/peer-review/10.1002/jbm4.10744.

## Supporting information


**Supplemental Table S1.** Clinical Research Questions Considered for Literature Search and Drafting the Statements
**Supplemental Table S2.** MeSH and Free Text Terms Used for Literature Search
