# Supplementary material for: Asia‐Pacific Consensus Recommendations on X‐Linked Hypophosphatemia: Diagnosis, Multidisciplinary Management, and Transition From Pediatric to Adult Care
Source: JBMR Plus. 2023 May 1;7(6):e10744. doi: 10.1002/jbm4.10744 (PMC10241092; doi:10.1002/jbm4.10744)
Supplement: Supplementary file 1 — Supplemental Table S1. Clinical Research Questions Considered for Literature Search and Drafting the Statements Supplemental Table S2. MeSH and Free Text Terms Used for Literature Search [file JBM4-7-e10744-s001.docx]

**Table S1.** Clinical research questions considered for literature search and drafting the statements.

| **Question no.** | **Clinical research questions** |
| --- | --- |
| **Screening and diagnosis** | |
| 1 | What are the red flags pertaining to clinical presentation that should prompt the conduction of investigations for renal phosphate wasting conditions in children and adults? |
| 2 | What are the presumptive criteria (clinical, biochemical, radiological) that should be considered to confirm the diagnosis of XLH in children and adults, based on the resources available in APAC settings? |
| 3 | What are the confirmative diagnostic criteria (clinical, biochemical, radiological, genetic) for XLH in children and adults? |
| **Multidisciplinary management and care** | |
| 4 | In APAC settings, what should be the goals for optimal multidisciplinary management of XLH in children, adolescents, and adults, and during the transition phase (from children/adolescents to adults)? |
| 5 | According to the latest international guidelines/consensus, the current approved medical therapy for XLH includes a combination of oral phosphate and active vitamin D (calcitriol or alfacalcidol) (conventional therapy), or Burosumab along with correction of vitamin D deficiency using native vitamin D supplements (cholecalciferol or ergocalciferol). Can these recommendations be broadly applied to APAC settings?   - What are the treatments available in each participating country? - Are there any specific constraints that limit the applicability of international treatment recommendations to APAC settings? |
| 6 | In APAC settings, what are the various specialties, a treating clinician *(family physician/primary care specialist, GP, pediatrician, endocrinologist, nephrologist, or treating clinician from any other specialty)* can refer a child diagnosed with XLH to, for holistic multidisciplinary care of XLH? |
| 7 | In APAC settings, what are the various specialties, a treating clinician *(family physician, general practitioner, endocrinologist, nephrologist, or treating clinician from any other specialty)* can refer an adult diagnosed with XLH to, for holistic multidisciplinary care of XLH? |
| 8–10 | What should be the recommended follow-up monitoring assessments and their frequencies to ensure optimized multidisciplinary care of children, adolescents and adults with XLH in APAC settings? |
| 11 | What should be the recommended additional (other than those recommended for adults)  follow-up assessments and their frequencies, and changes in conventional medical treatment for XLH in the following special populations: Pregnant and lactating women, menopausal women, and surgical patients? |
| 12 | What should be the recommendations to integrate telemedicine or digital medicine platforms for enhanced and continued follow-up and multidisciplinary care of XLH patients in APAC settings? |
| **Transition from pediatric to adult care** | |
| 13 | What should be the age (range) of XLH patients at which the treating clinician should initiate talks about transition of care with the child/family?   - What should be the optimal age of XLH patients at which the treating clinician should start transition of care? |
| 14 | What should be the roles and responsibilities of various stakeholders (patients, caregivers, pediatric and adult XLH experts, patient advocacy groups) to ensure successful transition of care of XLH from childhood to adulthood in APAC settings?   - - How can we assess XLH patients and caregivers to ensure their readiness for transition of care?   - What is the role of transition clinics in ensuring optimal transition of care? |
| 15 | What should be the checklist of information that should be passed on from the pediatric XLH expert to adult XLH expert during the transition of care? |
| **Education and training** | |
| 16 | What should be the educational/training initiatives that pediatric and adult XLH experts can undertake to develop or optimize multidisciplinary care of XLH in APAC settings (especially in settings with lack of access to XLH experts)? |

*XLH, X-linked hypophosphatemia; APAC, Asia-Pacific*

**Table S2.** MeSH and free text terms used for literature search.

|  | **Search terms** |
| --- | --- |
| MeSH terms | X-linked hypophosphatemia; rickets, X-linked hypophosphatemic; vitamin D-resistant rickets, X-linked; vitamin D-resistant rickets, hereditary; familial hypophosphatemic rickets; osteomalacia; rare diseases; orphan diseases; pregnan*; lactati*; breast feeding; menopause; surgical procedure, operative; operative procedures; transition of care; patient transition; care transition; patient transfer; referral and consultation; referral; telemedicine; telehealth; mobile health; checklist |
| Free text terms | Renal phosphate wasting; renal phosphate leak; surgery |
